# *In silico* analysis and theratyping of an ultra-rare CFTR genotype (W57G/A234D) in primary human rectal and nasal epithelial cells

**DOI:** 10.1016/j.isci.2023.108180

**Published:** 2023-10-12

**Authors:** Karina Kleinfelder, Virginia Lotti, Adriana Eramo, Felice Amato, Stefania Lo Cicero, Germana Castelli, Francesca Spadaro, Alessia Farinazzo, Daniele Dell’Orco, Sara Preato, Jessica Conti, Luca Rodella, Francesco Tomba, Angelo Cerofolini, Elena Baldisseri, Marina Bertini, Sonia Volpi, Valeria Rachela Villella, Speranza Esposito, Immacolata Zollo, Giuseppe Castaldo, Carlo Laudanna, Eric J. Sorsher, Jeong Hong, Disha Joshi, Garry Cutting, Marco Lucarelli, Paola Melotti, Claudio Sorio

**Affiliations:** 1 Department of Medicine, University of Verona, Division of General Pathology, 37134 Verona, Italy; 2 Department of Oncology and Molecular Medicine, Istituto Superiore di Sanità, 00161 Rome, Italy; 3 Department of Molecular Medicine and Medical Biotechnology, University of Naples Federico II, 80131 Naples, Italy; 4 CEINGE-Biotecnologie Avanzate Franco Salvatore S.c.a.r.l., 80145 Naples, Italy; 5 Confocal Microscopy Unit, Core Facilities, Istituto Superiore di Sanità, 00161 Rome, Italy; 6 Department of Neurosciences, Biomedicine and Movement Sciences, Section of Biological Chemistry, University of Verona, 37134 Verona, Italy; 7 Endoscopic Surgery Unit, Azienda Ospedaliera Universitaria Integrata Verona, 37126 Verona, Italy; 8 Cystic Fibrosis Centre, Azienda Ospedaliera Universitaria Integrata Verona, 37126 Verona, Italy; 9 Department of Pediatrics, Division of Pulmonary, Allergy/Immunology, Cystic Fibrosis & Sleep, Emory University, Atlanta, GA 30322, USA; 10 McKusick-Nathans Department of Genetic Medicine, Johns Hopkins University School of Medicine, Baltimore, MD 21287, USA; 11 Department of Experimental Medicine, Sapienza University of Rome, 00185 Rome, Italy; 12 Pasteur Institute, Cenci Bolognetti Foundation, Sapienza University of Rome, 00161 Rome, Italy

**Keywords:** Therapy, Human specimen, Molecular medicine, Integrative aspects of cell biology, Pharmacoinformatics, In silico biology

## Abstract

Mutation targeted therapy in cystic fibrosis (CF) is still not eligible for all CF subjects, especially for cases carrying rare variants such as the CFTR genotype W57G/A234D (c.169T>G/c.701C>A). We performed *in silico* analysis of the effects of these variants on protein stability, which we functionally characterized using colonoids and reprogrammed nasal epithelial cells. The effect of mutations on cystic fibrosis transmembrane conductance regulator (CFTR) protein was analyzed by western blotting, forskolin-induced swelling (FIS), and Ussing chamber analysis. We detected a residual CFTR function that increases following treatment with the CFTR modulators VX661±VX445±VX770, correlates among models, and is associated with increased CFTR protein levels following treatment with CFTR correctors. *In vivo* treatment with VX770 reduced sweat chloride concentration to non-CF levels, increased the number of CFTR-dependent sweat droplets, and induced a 6% absolute increase in predicted FEV1% after 27 weeks of treatment indicating the relevance of theratyping with patient-derived cells in CF.

## Highlights

• Very rare CFTR variants/complex genotypes should be studied as N-of-1 case
• Theratyping in patient-derived nasal and colon cells provides overlapping results
• FIS and Ussing chamber assays provide complementary information on CFTR function
• A234D variant is responsible for the response to CFTR modulators

## Graphical abstract

**Figure:**
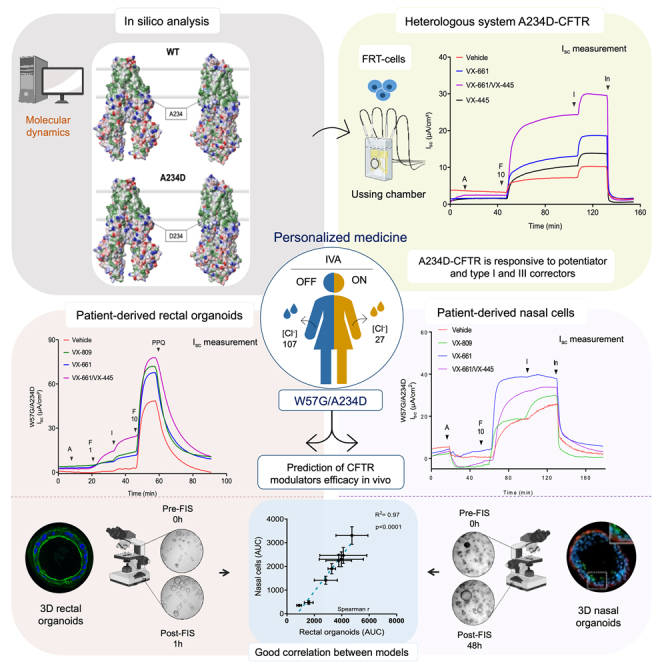

## Introduction

Cystic fibrosis (CF) is the most common severe autosomal recessive disorder in Europe, with a prevalence of 1 in 3000–4000 newborns in the Caucasian population.^1^ CF is caused by mutations in the cystic fibrosis transmembrane conductance regulator (CFTR) gene that is located on chromosome 7.^2^ It encodes an ATP-binding cassette transporter ion channel,^3^ exerting a key role in both chloride (Cl^−^) and bicarbonate (HCO_3_^−^) conductance through epithelia and in the regulation of other transporters and ion channels.^4^^,^^5^ The aberrant CFTR anion secretion affects lungs, pancreas, and other organs, resulting in the production of thickened mucus with chronic lung infections and pancreatic insufficiency.^1^^,^^6^^,^^7^

CF therapies (mucolytics, antibiotics, bronchodilators, anti-inflammatories, nutrition therapies, etc.) are usually intended to treat symptoms in order to improve CF patients’ quality of life. Compounds targeting specific defects in the CFTR protein, called CFTR modulators, have been developed to treat mainly subgroups of CF patients carrying the most prevalent CFTR mutations.^8^^,^^9^ To date, over 2000 different CFTR variants have been identified, among which 719 are known to cause CF (www.CFTR2.org, accessed on August 10^th^, 2023). They are grouped into six (with a seventh one proposed) functional classes according to the mechanism by which they alter the expression and function of CFTR protein.^10^^,^^11^ The VX661-VX445-VX770 (elexacaftor–tezacaftor–ivacaftor) triple combination is effective in up to 78% of patients carrying F508del/minimal genotypes with a satisfactory safety profile.^12^ However, for rare variants such as the ones of the subject of this study, only a few patients can potentially be recruited worldwide, and the lack of a relevant cohort of subjects sharing at least one common mutation makes it virtually impossible to carry out clinical trials with a classic design.

The concept of “theratyping” (identification of variants that are responsive to certain CFTR modulators)^13^^,^^14^ has recently been used to continuously update the original classes.^11^ It uses the rationale that variants belonging to the same class are treatable with the same therapy and should respond similarly to the used compound.^15^ Despite the usefulness of this strategy to rationalize therapies, the biological effect of the majority of CFTR variants remains unclear, mainly for rare or unclassified variants. Moreover, the same response to modulators is not always reported for variants belonging to the same class,^16^ or even for the same variant in different individuals.^16^ Thus, the development of new drugs must proceed with increasingly sensitive, predictive, and precise monitoring biomarkers, allowing the best possible selection of treatment for individual patients.

The strategy for personalized medicine includes the use of patient-derived intestinal organoids,^17^^,^^18^^,^^19^ leukocytes,^20^^,^^21^ and human nasal epithelial cells.^22^ The use of rectal biopsies to guide the diagnosis of difficult cases has a long history in CF,^23^^,^^24^ showing the relevance of this epithelium as a robust indicator of CFTR function. Another issue is represented by the predictive value of respiratory systems-derived cells, as they appear suitable models to reproduce the defect in CFTR function, as well.^25^^,^^26^ Interestingly a side-by-side comparison of data in the same patients is just beginning to emerge^27^^,^^28^^,^^29^ and recent data point to a better performance of intestinal epithelial cells over nasal-derived cells for preclinical drug testing applications.^30^ In this report, we describe the response to available CFTR-targeted drugs of a combination of ultra-rare variants described in the following section. The aim of our work was to theratype a patient with CFTR genotype W57G/A234D through a detailed analysis and comparison of the response of her upper airways (nasal cells) and intestinal organoids to available CFTR modulators. Data on the response of A234D variant (both untreated and treated with CFTR modulators) expressed in FRT (Fisher rat thyroid) and CFBE (cystic fibrosis bronchial epithelial) cells are also shown. The treatment of this patient with ivacaftor confirmed the predictive value of theratyping testified by the rapid and significant improvement of functional tests and clinical condition.

## Results

### W57 and A234 are located in different structural regions of CFTR and their mutation is predicted to destabilize the channel conformation to a different extent

Given the limited information available for the variants W57G and A234D, we performed *in silico* analysis of the predicted structure of these variants in comparison with the native sequence. Binding of ATP to the nucleotide-binding domains of CFTR drives a rearrangement of the transmembrane helices, resulting in a major conformational change, which serves to open a gate to the transmembrane flow of Cl^−^ anions down their electrochemical gradient. Very recently, the cryoelectron microscopy structure of human CFTR in its ATP-free, unphosphorylated open conformation and ATP-complexed closed conformation became available^31^^,^^32^ thus allowing unprecedented insights into the molecular features of CFTR at the atomic detail of resolution. The binding of VX770 at the protein-lipid interface in the transmembrane region has been shown not to significantly alter the three-dimensional structure of the channel.^32^ Alternative sites for the binding of VX770 to CFTR have been recently suggested based on molecular dynamics simulations^33^ and molecular docking.^34^ In this work, we focused on the highest-resolution structural information, which defined a specific binding site in CFTR for VX770 and used this structure to assess the potential effect of the observed point mutations on the CFTR structural stability and to infer possible functional consequences of the amino acid substitutions.

**Figure 1:**
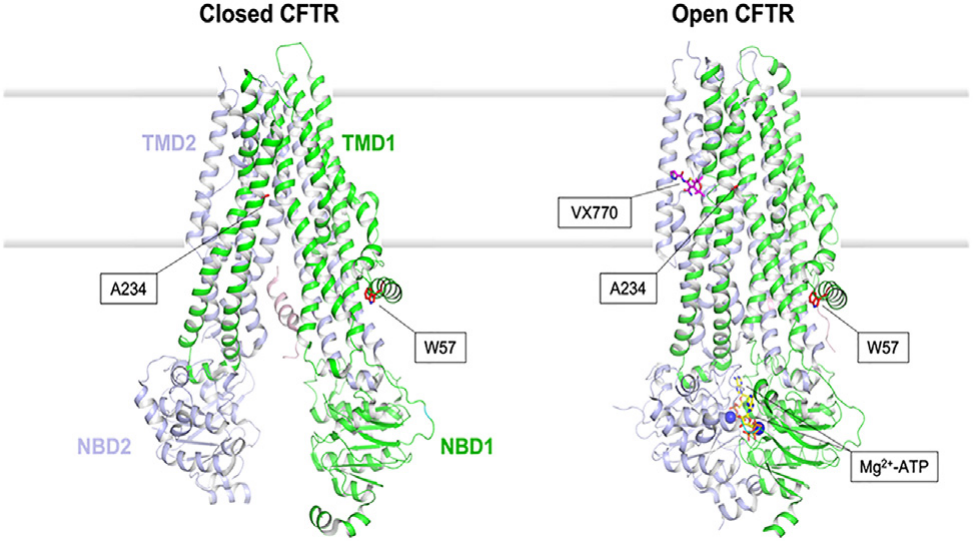
Structure of CFTR in its ATP-free, unphosphorylated closed conformation and ATP-bound open conformation, in complex with the VX770 (ivacaftor) potentiator Transmembrane domains (TMDs) and nucleotide-binding domains (NBDs) are represented by green and slate cartoons and labeled. The residues mutated in this study (W57 and A234) are represented by red sticks and labeled. VX770 and Mg^2+^-ATP are represented by magenta and yellow sticks, respectively (cations by blue spheres), and labeled. The membrane bilayer is represented by shaded thick gray lines. The structural models have been obtained as explained in the methods section, using the 5UAK (closed CFTR) and 6O1V (open CFTR) pdb files as inputs.

Figure 1 shows the location of the two residues mutated in the present study. Trp57 is located in the lasso motif on the cytosolic side of the channel and forms a compact interface with the TMD1 domain. The indole group of W57 is mostly buried in the protein milieu in the closed conformation (side-chain accessibility of 23.2%); in the open state, the burial becomes even more prominent (side-chain accessibility of 8.8%). Overall, W57 contributes to local hydrophobic surface patches with a mild contribution to a positively charged surface patch, attributable to the amine in the indole group, partly solvent-exposed in the closed CFTR conformation.

**Figure 2:**
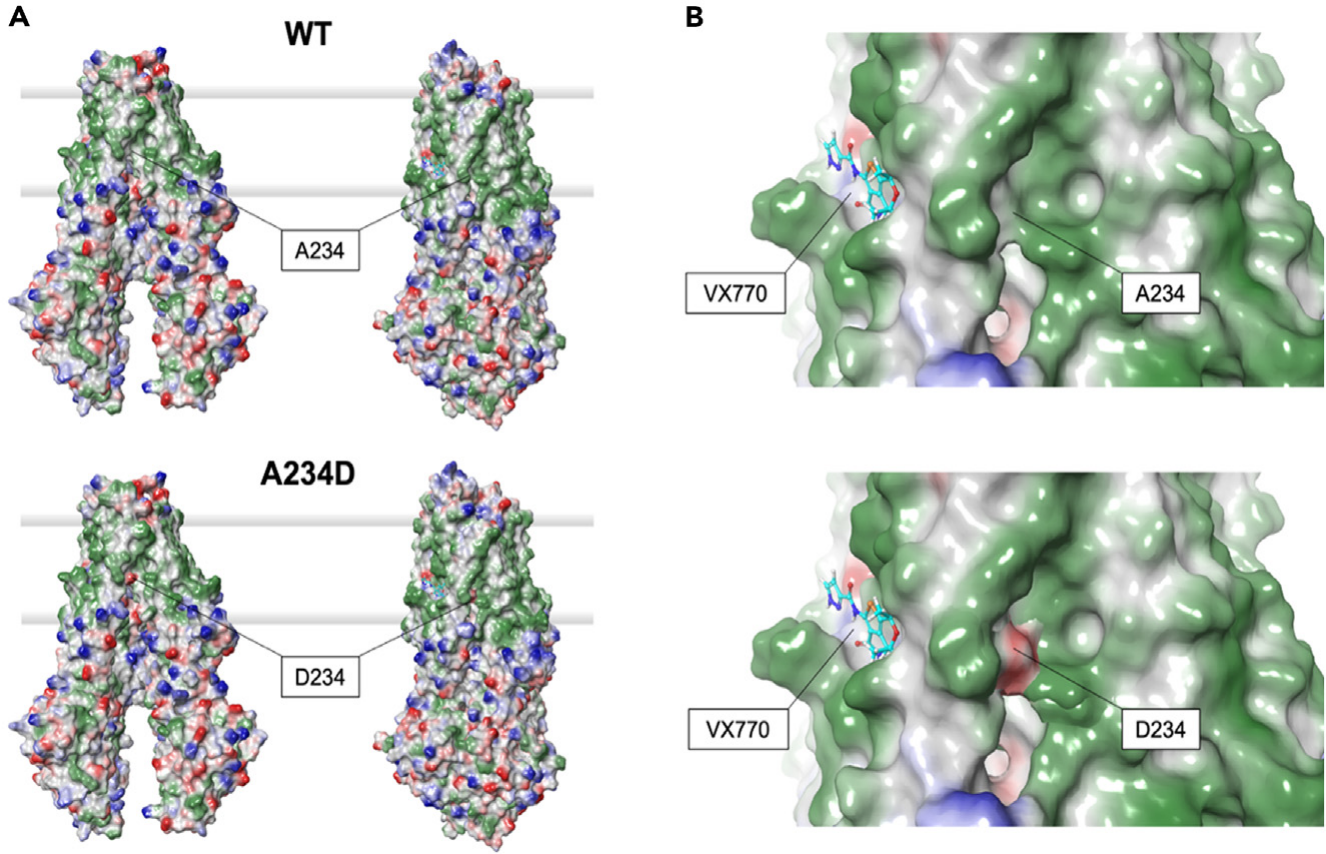
Molecular surface properties of CFTR (A) Comparison of the molecular surface of CFTR in its closed (left) and open (right) conformations. WT (top) and A234D variant (bottom) are compared. Green patches represent hydrophobic surface and blue and red patches represent positive and negative electrostatic patches, respectively. The membrane bilayer is represented by shaded thick gray lines. (B) Zoom on the VX770 binding cavity region in WT (top) and A234D (bottom) CFTR in its open conformation. VX770 is represented by sticks, C atoms are in cerulean blue, O atoms in red, N atoms in dark blue, S in yellow. The color scheme of surface patches is the same as in (A).

The other mutated residue, A234, is located in TMD1 in helix 4, and its methyl side chain is similarly exposed to the membrane in both the closed (32.6%) and open (35.4%) CFTR conformations. By protruding both toward helix 6 and the membrane milieu, the side chain of A234 contributes to local hydrophobic surface patches in both cases (Figures 2A and 2B top panels). Interestingly, the location of A234 is relatively close to the binding pocket of VX770, mostly formed by the helix-loop transition of transmembrane helices 8 and 5, flanked by helix 4 (Figure 1, right; Figure 2B, top).

We performed *in silico* mutagenesis to assess the effects of W57G and A234D substitutions on the structural stability of CFTR in its closed and open conformations. The change in protein stability was quantified by computing the ΔΔGapp descriptor, expressing the difference in kilocalories per mole between the free energy of folding of the variant and that of the wild type (WT) (ΔΔGapp = ΔGappmut – ΔGappWT; see STAR Methods section). Both mutations were predicted to destabilize either conformation of CFTR, though to a different extent. In particular, A234D was predicted to destabilize the closed conformation (ΔΔGapp = 4.63 kcal/mol) less than the VX770-bound open conformation (ΔΔGapp = 16.80 kcal/mol). A much stronger destabilizing effect was predicted for the W57G substitution, which dramatically affects both the stability of the closed and open CFTR conformations to a similar extent (ΔΔGapp = 28.40 and 27.04 kcal/mol, respectively). Such highly destabilizing effects are attributable to the loss of key hydrophobic interactions that would strongly perturb the tight interaction between the lasso motif and the TMD1 helices. Conversely, the introduction of a carboxy group by the A234D substitution in helix 6 creates a negatively charged surface patch within the transmembrane domain (Figures 2A and 2B), which is evidently more detrimental for the open CFTR conformation. However, the binding pocket of VX770 would be substantially unperturbed by the substitution (Figure 2B).

### W57G/A234D CFTR function and modulator efficacy in 3D nasal organoids and colonoids

**Figure 3:**
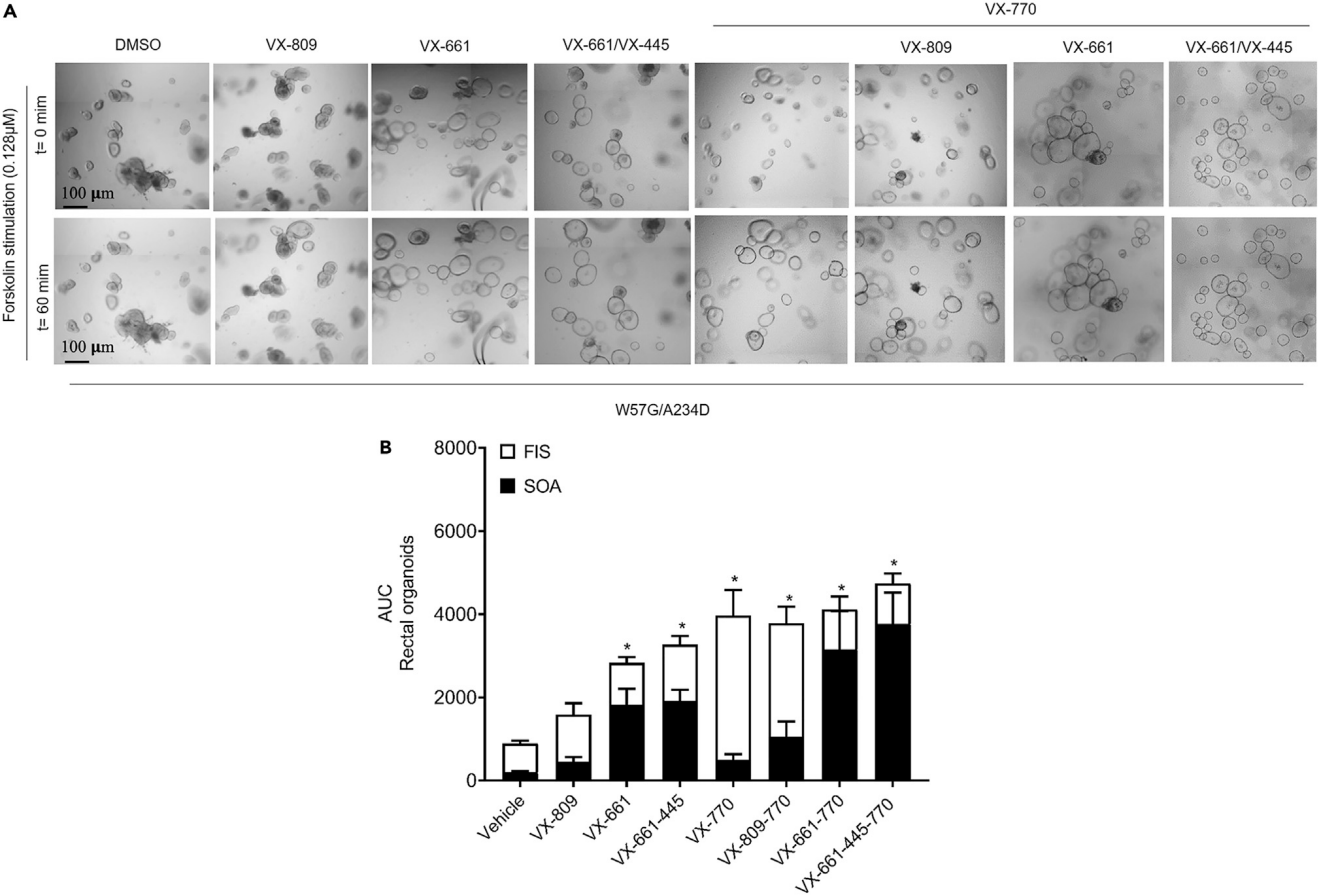
Pharmacological correction of CFTR function in W57G/A234D rectal OGs measured by the FIS assay (A) Representative bright-field images of W57G/A234D organoids stimulated with 0.128 μM of FSK with or without CFTR modulator treatment, as indicated. (B) Quantification of rectal OGs swelling preincubated for 24 h with the vehicle (DMSO 0.1% v/v) and the CFTR correctors (VX809-661-445 individually or in combination as indicated). At the time of the assay, rectal OGs were stimulated with various concentrations of forskolin (data selected from 60 min at 0.128 μM forskolin +VX770 3 μM). Black bars represent the value of steady-state total organoid area (SOA) before the addition of forskolin. White bar is the total organoid area after addition of forskolin (FIS assay). Ordinary one-way ANOVA: ∗< 0.05; ∗∗p < 0.002; ∗∗∗p < 0.0002; ∗∗∗∗p < 00001.

It has previously been demonstrated that CFTR-mediated Cl^−^ secretion measurement in rectal biopsies is a reliable predictive biomarker for both the prognosis and diagnosis of CF^23^ and a robust method to assess the *in vivo* efficacy of CFTR modulators in CF patients undergoing *in vivo* treatment.^35^^,^^36^ However, its application as a model to test the efficacy of exogenously applied, slow-acting CFTR correctors is hampered by the gradual loss of tissue viability under *ex vivo* incubation conditions (within hours rather than days). To assess the *ex vivo* efficacy of the CFTR residual function and CFTR modulators, we used the forskolin-induced swelling (FIS) assay in intestinal organoids as a potential biomarker and predictor of the clinical benefit for these compounds.^37^^,^^38^ First, the CFTR residual function was detected in the W57G/A234D organoids upon forskolin treatment within a range of 0.02–5 μM. To test the CFTR modulator responses, the organoids were preincubated for 24 h with correctors (VX809 lumacaftor, VX661 tezacaftor, and/or VX445 elexacaftor) and acutely stimulated with forskolin alone or in combination with the potentiator (VX770), using DMSO as a vehicle control. The outcome showed that VX770 treatment, either alone or in combination with correctors, increased organoid swelling throughout the whole range of forskolin concentrations tested (Figures 3A, 3B, and S1). The FIS rates in those organoids preincubated with VX661 and VX661 + VX445 were negatively affected by their partially recovered steady-state lumen area prior to forskolin treatment, leading to an underestimation of the effect of VX661 alone or together with VX445 on restoring CFTR function. This effect is highlighted by the apparently higher FIS for rectal organoid cultures treated with VX809 + VX770, since VX809 did not cause variations in the luminal size prior to forskolin stimulation in our setting (Figures 3 and S1), as was also reported in other studies.^19^^,^^37^

**Figure 4:**
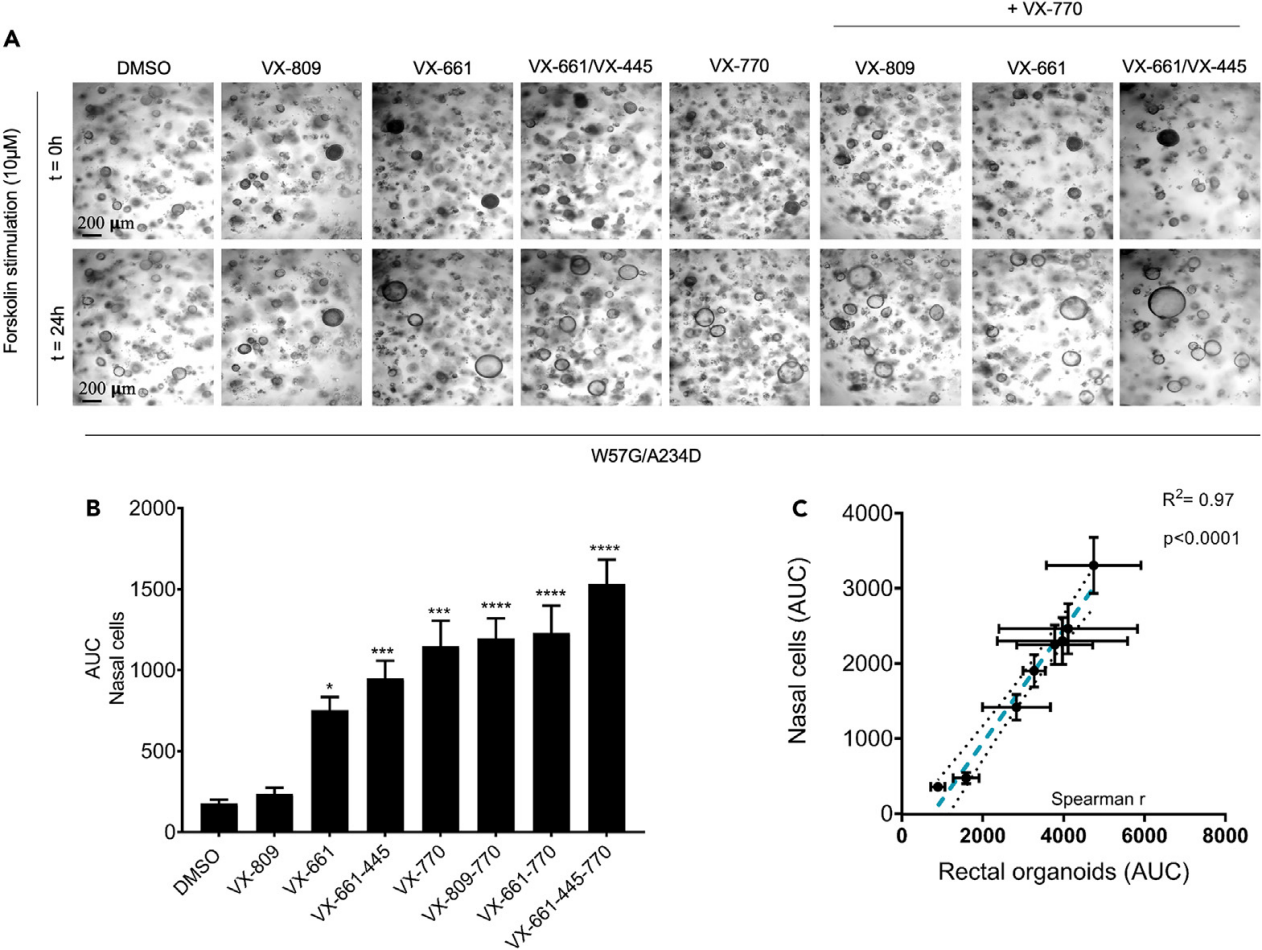
FIS assay of W57G/A234D nasal organoids (A) Representative bright-field images of W57G/A234D nasal organoids stimulated with 10 μM of FSK with or without CFTR modulator treatment, as indicated. (B) Quantification of FIS rates after the addition of 10 μM forskolin + 5 μM VX770 on 48 h pre-treated nasal organoids with indicated CFTR modulators. (C) Correlation plot of AUC (X ax Colon and Y ax Nasal organoids, R2 = 097, p < 0.0001). Dashed blue line represents best-fit values. The two black dotted lines represent 95% confidence interval of the best-fit line. Ordinary one-way ANOVA: ∗< 0.05; ∗∗p < 0.002; ∗∗∗p < 0.0002; ∗∗∗∗p < 00001.

Taking in consideration the pharmacological recovery of organoids area treated with VX661 and VX661 + VX445 in absence of forskolin is now possible to appreciate the corrective effect of these molecules on W57G/A234D 3D rectal organoids (OGs). Indeed, the total swelling of VX661 and VX661 + VX445-treated organoids in FIS with the addition of drug rescued steady-state organoid area (SOA) becomes similar to area under the curve (AUC) values of swollen rectal OGs treated with VX770 and/or VX809 + VX770 in response to forskolin (Figure 4A).

The positive effect of VX661 and VX661+VX445 in improving the activity of the W57G/A234D-CFTR channel was also observed in FIS done in nasal epithelial cells that did not demonstrate the pre-swollen phenomena seen in rectal OGs due to the addition of these correctors (Figures 4A and 4B). Similarly done for rectal organoids, the characterization of dose response was performed also for 3D nasal organoids, for some of the pharmacological condition tested, in order to determine the magnitude of swelling for W57G/A234D nasal organoids, using increasing doses of stimulatory forskolin (0.128–10 μM) (Figure S2). Additionally, as unsynchronized organoid differentiation reduces the accuracy of quantifying FIS for nasal organoids,^39^ we performed immunofluorescence experiments that demonstrate 3D nasal organoids stained positive for mucin 5B, acetylated α-tubulin, cytokeratin 5 and CFTR, as well as the visual observation of beating cilia, confirming the phenotype of differentiated organoids (Figure S3 and Video S1). Moreover, the increase of swelling levels due to improvement of CFTR function rescued by VX809, VX661, and VX661 + VX445 alone or in combination with VX770 (ivacaftor) in nasal cells correlates well with the total swelling registered in 3D rectal OGs (Figure 4C). For this comparison, we selected the concentration of 0.128 μM of forskolin used in rectal OGs, which does not saturate FIS, and of 10 μM for nasal cells because it induces the highest FIS rates specifically in modulator-treated organoids; and best correlates with clinical outcomes, i.e., *in vivo* biomarkers of CFTR function.^40^^,^^41^

Document S1. Figures S1–S4 and Table S1

### W57G/A234D CFTR function and effect of modulators in nasal and colonoid-derived 2D monolayers

**Figure 5:**
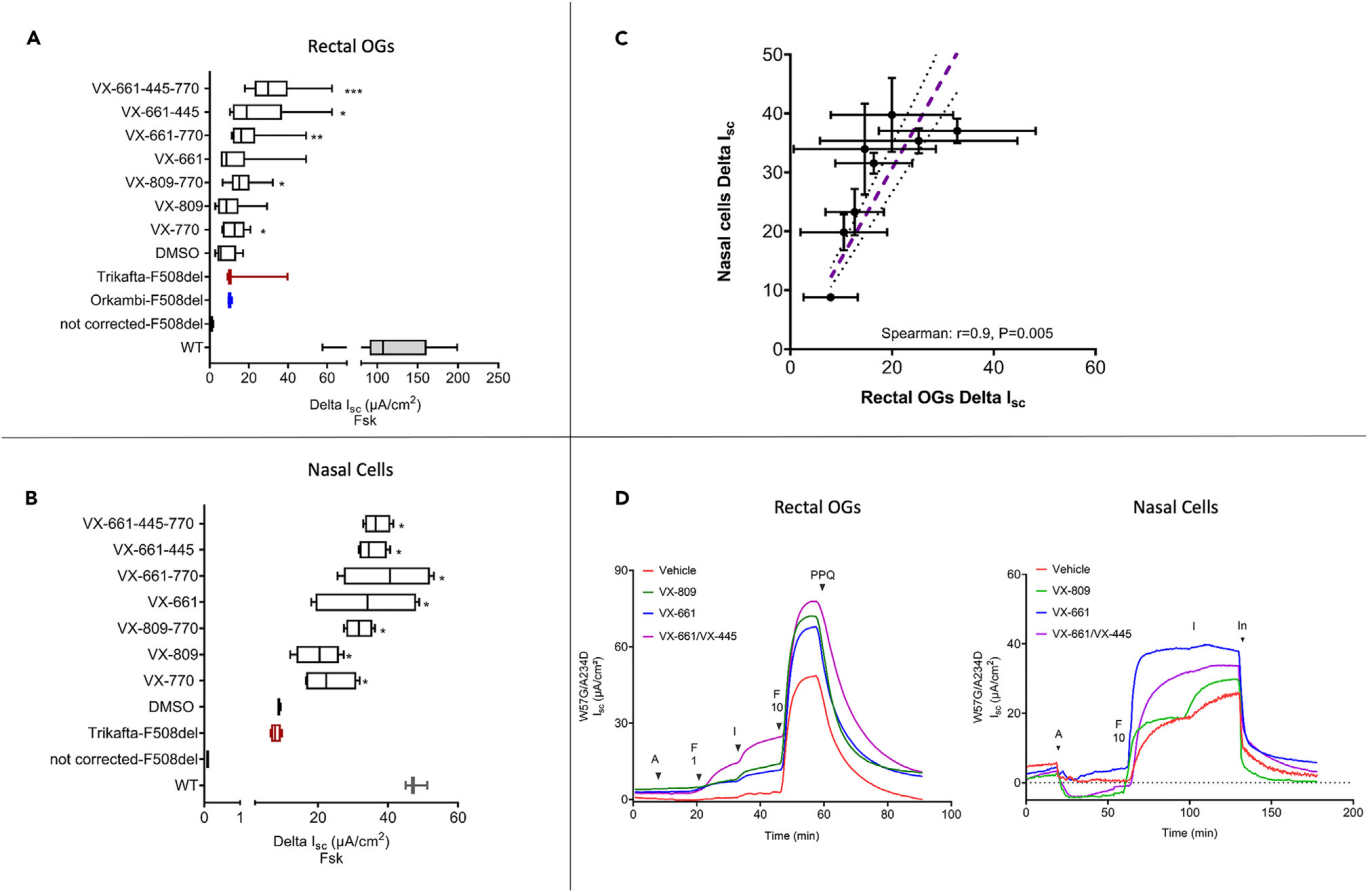
Functional characterization of W57G/A234D CFTR by short-circuit current experiments (A) Rectal OGs growing as 2D monolayer. The data are expressed as Delta Isc values (based on maximal fsk response) of different treatments (*p* was calculated with the Kruskal-Wallis test). (B) Delta Isc values expressed as μA/cm^2^ of monolayers of CRC-differentiated nasal cells. Cells were treated with or without VX809 (3 μM), VX661 (3 μM), VX445 (2 μM), and combinations as indicated for 24 h with DMSO as the mock control. (C) Spearman correlation of the Delta Isc values measured in the same samples present in panels A and B showing an excellent correlation among measure values in the two models. Dashed pink line represents best-fit values. The two black dotted lines represent 95% confidence interval of the best-fit line. (D) Representative tracings of monolayers derived from rectal OGs and CRC-differentiated nasal cells (A: Amiloride 100 μM F1: FSK 1 μM, I: VX770 1μM, F10: FSK 10 μM, In: PPQ-102 20 μM (rectal OGs) or Inh-172 (nasal cells) both at 10 μM). Isc values from WT and corrected-F508del and not corrected-F508del were used here as reference.

To confirm the functional consequence of the W57G/A234D CFTR genotype, we assessed the transepithelial ion transport in a polarized monolayer derived from 3D intestinal organoids and mounted in Ussing chambers. The addition of forskolin elicited a chloride secretory response in the W57G/A234D monolayers, confirming a residual function of this CF genotype. For intestinal organoids, we were able to calculate its activity that corresponded approximately 7% of healthy controls (WT) that was further potentiated by the acute addition of VX770 (to approximately 12% of WT) and completely inhibited by the CFTR inhibitor PPQ-102, confirming its CFTR dependency. Moreover, consistently with the data obtained from the FIS assay, upon overnight incubation with correctors (VX809 or VX661 alone or in combination with VX445), the CFTR-associated current displayed an increasingly greater magnitude compared to the vehicle-treated one, reaching a maximum of approximately 24% of the WT (Figure 5A), suggesting the capability of these compounds to increase the amount of activatable (or partially active) CFTR. A statistically significant difference in corrector efficacy was detectable with all the combinations that include VX661 and VX770 alone. We performed the same procedure with conditionally reprogrammed cell (CRC)-derived nasal cells with an overlapping result (Figure 5B). Interestingly, there is an excellent concordance between the responses recorded in nasal and intestinal-derived cells (Figure 5C, C: Spearman r = 0.9, p = 0.005).

### W57G/A234D CFTR native protein expression and processing rescue by correctors in nasal epithelial cells (ALI-cultures) and intestinal organoids

**Figure 6:**
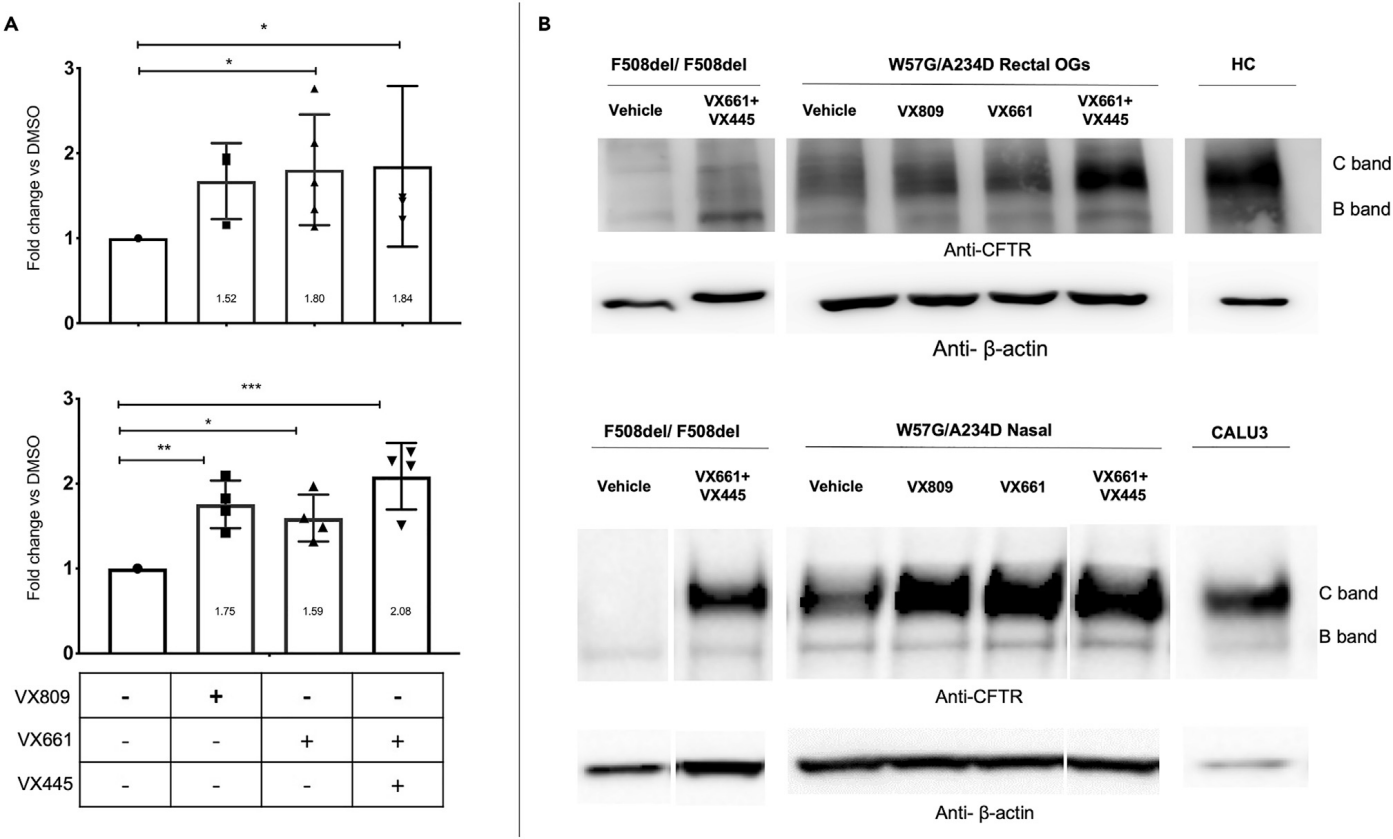
Expression of CFTR protein in W57G/A234D by western blot analysis Rectal OGs (upper panel), nasal epithelial cells (lower panel). Non-CF controls are made of intestinal organoids (HC) or Calu3 cells, respectively. (A) Densitometric analysis of the results shown as representative western blots in (B). The controls show the presence of C and B bands related to the different CFTR glycosylation states. F508del/F508del samples were used as a reference and show an increase in the expression of B and C bands following treatment with a combination of VX661 with VX445. The newly synthesized non-glycosylated polypeptidic chain (band B) is expressed at variable levels in W57G/A234D colonoids. Treatment for 24 h of intestinal colonoids with CFTR modulator VX809, VX661 alone, or in combination with VX445 improved the expression of band C reaching statistical significance. In VX809-treated rectal OGs, the increased expression almost reaches statistical significance (p = 0.06). An overlapping trend of drug-induced CFTR Band C increase was observed in nasal cells. β-actin polyclonal antibody was used as a loading control. The results of the densitometric analysis are shown in (A), summarize the β-actin-normalized data (C + B bands) from a minimum of three independent experiments. ∗p < 0.05, ∗∗p < 0.005 and ∗∗∗p < 0.002 (ANOVA and Kruskal–Wallis tests).

The functional data suggest the presence of a protein with a residual function that can be further rescued using CFTR modulators. To explore how the W57G/A234D CFTR genotype affects CFTR processing, the protein extracts obtained from intestinal organoids were analyzed by western blot (WB) to detect the presence of immature (band B) and/or mature (band C) forms of CFTR protein. As shown in Figure 6, the CFTR protein expressed by the W57G/A234D CFTR variants was detectable in unprocessed cells. The higher MW band (labeled band C) runs within the range of the fully glycosylated healthy control, while a lower band (labeled band B) runs at a lower apparent MW than the mature, core-glycosylated band. In line with the functional data, after treatment with all the correctors utilized, an increased level of CFTR protein rescue was detected, reaching statistical significance with the treatments of VX661 alone and VX445 + VX661 providing the highest level of protein expression. Nevertheless, the expression levels of both band B and C appeared significantly lower than those of the WT organoids, as expected from a CF patient and in line with the detection of a residual CFTR function in untreated organoids (Figures 3 and 4). The same analysis was performed in ALI cultures of nasal epithelial cells from the same patient with overlapping results (Figure 5). Based on the average values derived from the densitometric analysis of WB data, the treatment with all correctors and combination appear to induce a significant increase in CFTR protein expression relative to untreated samples, with a slight increase in protein expression for VX661 + VX445 and VX661 over VX809 (Figure 6). Evidence of substantial increase of mature form of CFTR protein upon treatment with VX661 + VX445 was also seen in nasal organoids growing in Matrigel in a 3D format (Figure 4 Suppl.).

### Concordance between assays

In Table 1, we summarize the results of the different assays, showing an overall concordance of the functional tests in defining the presence of residual CFTR activity supported by the presence of CFTR protein. The efficacy of all CFTR modulators appears to depend on the potentiation of the CFTR variant (identified as A234D based on published data that report essentially no response to CFTR modulators for the W57G variant and a moderate response for A234D variant) that appears to be sufficiently expressed at the cell membrane to support the effect of VX770. A further increase in protein expression is warranted by the effect of CFTR corrector VX809 and, to a higher extent by the combination of VX661 and VX445 + VX661, when we consider that both treatments induce a clear increase in SOA after 24 h incubation of colonoids, a condition that is well known to reduce the dynamic range of the FIS assay, leading to an underestimation of the effect when the AUC is calculated following the standard FIS assay. Altogether, we appreciate the superiority of VX661 and VX661 + VX445 toward VX809 as CFTR correctors but, based on the *in vitro* data, cannot support a clear preference between the first two combinations.

**Table 1:** Semi-quantitative evaluation of the response to different CFTR modulators and their combination

	SOA increase (rectal)	FIS (nasal + rectal)	Isc (nasal + rectal)	WB (nasal + rectal)
VX770	NA	++	+/−	NA
VX809	–	+/−	+/−	+
VX809 + VX770	NA	++	+/−	NA
VX661	+	+	+	+
VX661 + VX770	NA	+	+	NA
VX445 + VX661	+	+	++	++
VX445 + VX661 + VX770	NA	+	++	NA

No (−), very low (+/−), measurable (+), good (++) responses; Note that VX770 was added at the time of assay; SOA: steady-state organoid area; NA: not assayed; FIS: forskolin-induced swelling assay, Isc: short circuit currents (Ussing chamber), WB: western blot.

### Expression and theratyping of A234D variant in FRT and CFBE cells

**Figure 7:**
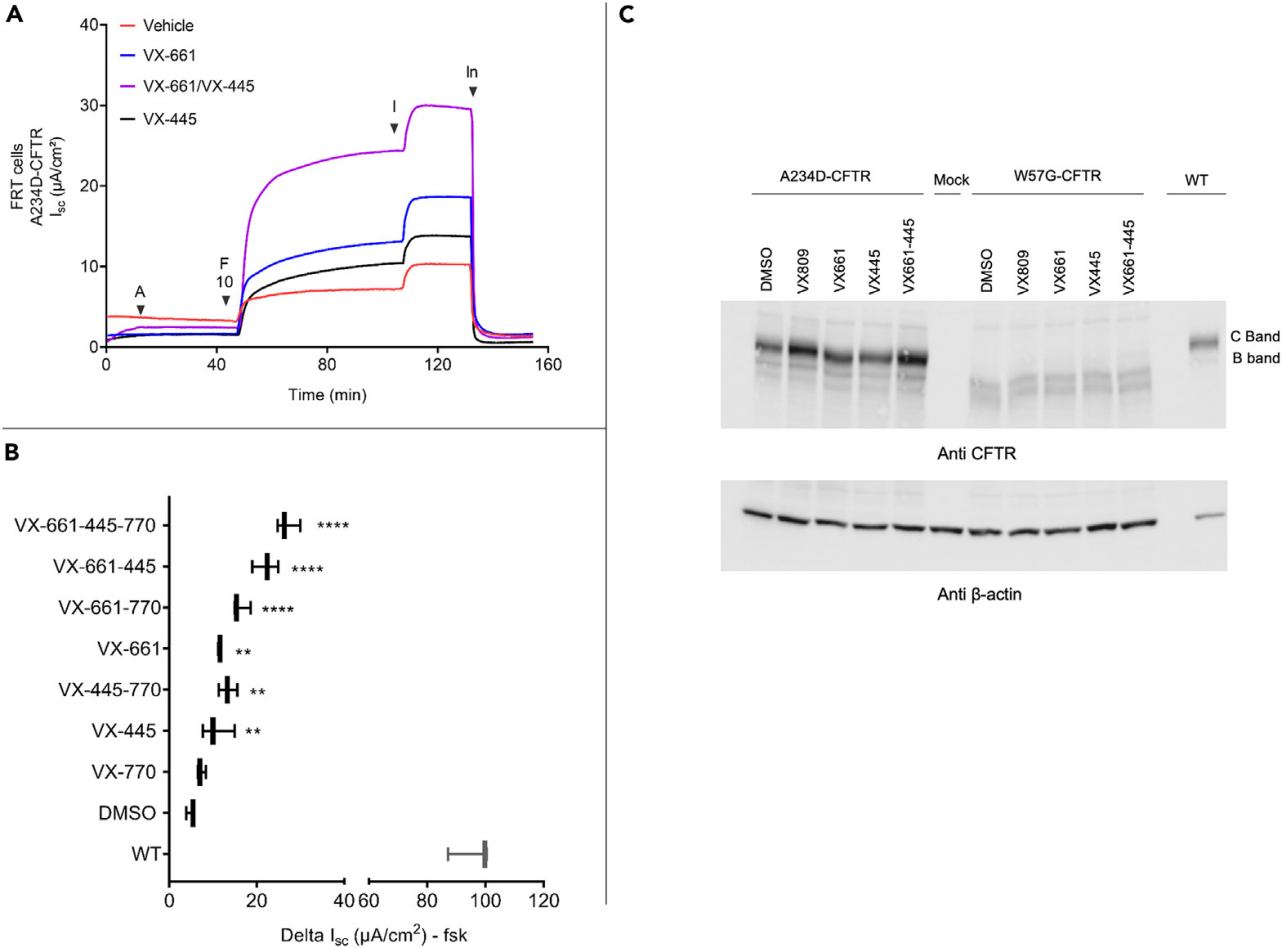
Expression of A234D-CFTR in FRT cells (A) Ussing chamber assay of FRT cells incubated for 24 h with VX445, VX661 (both at 3 μM), and their combination as indicated. Cells were then treated with amiloride 100 μM (A) followed by forskolin 10 μM (F10, both apical and basolateral side). VX770 (I) 5 μM and CFTRInh 172 (In) 10 μM, we next added to the apical side only in sequence. (B) Summary of the ΔIsc of three independent experiments. ∗∗p < 0.009 and ∗∗∗∗p < 0.0001 (Ordinary one-way ANOVA). (C) Representative western blotting image performed using A234D and W57G expressing FRT cells.

As previously discussed, the analysis of available literature indicates A234D as a candidate CFTR variant that accounts for the residual function and the response to CFTR modulators shown here. Although our goal is to theratype this individual patient, considering the limited data available in the literature, we performed theratyping of the A234D variant expressed in FRT cells. Figure 7 summarizes the data that complement clinical results obtained in this patient, i.e., the presence of a residual function (exhibiting approximately 7% of wild-type activity when tested in the FRT cell model) that is increased by treatment with the individual modulators VX445 and VX661. We also note a strong additive response when the two correctors were added together during the 24 h incubation (VX661 + VX445 + VX770 treatment achieved nearly 28% of wild-type function in FRT cells) as indicated by short circuit current measurement (Figures 7A and 7B). VX770 appears to increment the response in cells either untreated or treated with correctors. Altogether, these data confirm a key role of the A234D variant that accounts for the response detected *in vitro* in both nasal and intestinal primary cell models. Interestingly, the response to correctors was reproduced also in CFBE cells, although we measured a variable basal response in the clones (Table S1).

### Efficacy of Kalydeco treatment *in vivo*

The local authorities approved the treatment off-label with ivacaftor (VX770) and the patient was monitored during the treatment. As summarized in Table 2, the treatment was highly effective in reducing the chloride ion concentration in sweat already after seven days of treatment (which dropped from 112 to 36 and 23 mmol/L). The recovery was confirmed by the analysis of the sweat optical test (bubble test), where the number of sweat bubbles in the CFTR-dependent phase of the test substantially increased from complete absence before the treatment to clearly detectable values within 4 weeks from the beginning of the treatment. Furthermore, even if the lung function needs longer time frames to be properly evaluated, we measured an absolute change of a 4% improvement in FEV1% after 4 and 20 weeks of treatment, further increasing up to 6% after 27 weeks of this therapy. Altogether, these data indicate the efficacy of the treatment in the patient, in line with the *in vitro* data obtained using primary cells.

**Table 2:** *In vivo* response before and during treatment with VX770 (Kalydeco)

		GCST	Optical sweat bioassay of CFTR function^a^		Lung function
Weeks of treatment	Treatment	Cl^−^ (mmol/L)	Mean C/M ratio	C glands (n)	FEV1%
0	off	107-101/110–112^b^	0^b^	0^b^	64
1	on – Kalydeco (VX770)	36–35	0,0119	29	68
4	on – Kalydeco (VX770)	36–43	0,0696	81	NA
12	on – Kalydeco (VX770)	NA	NA	NA	65
20	on – Kalydeco (VX770)	23–27	NA	NA	68
27	on – Kalydeco (VX770)	NA	NA	NA	70

a Optical CFTR-dependent sweating test.^42^

b Results obtained at two different times; Sweat Cl^−^ values are reported for both forearms.

## Discussion

The aim of this study was to assess the efficacy of CFTR modulators on the W57G/A234D CFTR genotype using patient-derived cells models. Second aim was to compare the results of theratyping performed in different cell types derived from the same individual, i.e., colon and nasal-derived epithelial cells, and finally we characterized the response of the rare A234D variant expressed in FRT and CFBE cells. Our *in silico* modeling studies predicted that both W57G and A234D substitutions may destabilize the open and closed conformations of CFTR protein, but the magnitude of the effect is predicted to significantly depend on the variant. The W57G substitution dramatically destabilized both the closed and open CFTR conformations, while the A234D variant showed a milder destabilization of the closed state and a prominent destabilization of the VX770-bound, open conformation, which is predicted to be 10 kcal/mol less harsh than the one induced by the W57G substitution. Moreover, the structural analysis did not predict major alterations of the binding cleft of VX770 upon A234D substitution, which is compatible with the capability of the variant to still bind the potentiator. On the contrary, the major alteration in stability induced by the W57G substitution may prevent the correct switch of CFTR between its open and closed states, thus severely impairing the function of CFTR. The biochemical data indicate that the combination of these variants gives rise to a phenotype where CFTR is partially expressed. The observation of the presence of C-band and the detection of a residual function (detectable only at the higher FSK concentration of 5 μM) in untreated colonoids by the FIS assay, associated with a strong response to VX770, suggests a sufficient membrane localization to permit the activity of a CFTR potentiator.

The *in vitro* data on CFTR variants overexpressed in FRT cells or in human airway cell line report a very low residual function and response of the W57G variant to CFTR potentiators and correctors.^43^^,^^44^^,^^45^ Furthermore, an undetectable response for W57G and a response for A234D to 3 μM of FSK alone (estimated to be 17% of the wild type) with an increased response in the presence of VX770 (estimated to be 56% of the wild type) was reported.^46^ We confirmed these preliminary findings in FRT and CFBE cells where we observed residual function of the channel that is significantly improved by treatment with VX770 both in absence and/or presence of CFTR correctors. We can safely conclude that the response we detected was due to the A234D variant, which appears to have features of both a class II and III mutations. Of note is the fact that there is a concordance between the functional improvement detected by two different assays (FIS and Isc) following treatment with different drug combination and the increase in protein expression, as detected by WB in the presented case. The different response of the organoids to VX809 as compared to VX661 is not surprising, as recent data describe a dedifferentiation effect of VX809 + VX770 caused specifically by VX809, while VX661, a second-generation corrector developed based on VX809 structure, does not perturb the epithelial integrity of polarized bronchial epithelial monolayers.^47^ Of note, other authors have reported different mechanisms of action of the two molecules.^48^ The superior capability of VX661 to induce CFTR expression in this case can lead to reach a threshold of CFTR recovery sufficient to allow endogenous cAMP levels to re-establish the physiological ion exchange necessary to recover OG swelling to a level that is similar to milder phenotypes, as is evident by the clear presence of a filled lumen just after 24 of treatment with VX661 and VX661 + VX445 combinations.

This work can be viewed within a more general strategy aiming at the development of a personalized medicine approach in CF, possibly combining both *in silico* prediction and biochemical/functional data. Numerous *CFTR* variants have been functionally assessed in FRT, HEK-293, and CFBE cell lines, with the collection of valuable data but with the limitation of a different background relative to the cell type, the mechanism of expression (cDNA instead of whole gene), and variable expression levels, making cell models derived from the individual patient an ideal model for the evaluation of response, when available.

In our case, we detected in the colonoid model a corrector-induced swelling that modified the SOA without the addition of any cAMP agonist, indicating drug efficacy in correcting the phenotype; we could indeed measure a significant increase in the basal intestinal OG swelling following treatment with VX661 and VX661 + VX445 that does not occur when DMSO and VX809 are used. As the OG swelling before the FIS assay led to an underestimation of the drug efficacy using the standard FIS test, we have included the SOA value in the calculation to avoid a bias that might (wrongly) suggest a superior efficacy of VX809 in comparison with the other correctors utilized. Interestingly, no SOA in nasal CRC-derived organoids was noticed and the swelling developed only following stimulation with forskolin underlying a difference in the quality of response among models.

If we consider the available evidence present in the literature, we might consider that the sole capability to evaluate the drug response in patient-derived cells, even in absence of a clinically measurable response that is expected to occur in some circumstances, appears to be of great relevance also in view of different therapeutic options that might emerge in the near future and for which we must be prepared.

In this case, we presented that treatment with Kalydeco leads to a substantial improvement in *in vivo* CFTR function tests, as well as lung function, which was predicted by *in vitro* assays, suggesting that *ex vivo* models might represent powerful tools suitable for theratyping, of particular value for rare variants. Conclusive evidence will emerge following the constant stream of data coming from the international literature, accumulating evidence of this kind.^49^^,^^50^ Based on the *in vitro* data presented, we expect for this patient further improvement using other combinations that include CFTR correctors; however, additional drug prescription to the patient is currently not authorized by the local authorities, even though A234D has been approved for treatment with VX770/VX661/VX445 by the FDA in the U.S. Of note, approval for the clinical use of CFTR modulators for rare CFTR variants in the U.S. can be based on an *in vitro* assay of CFTR function alone when clinical trials are not available. This study followed the *n*-of-1 trial concept, which is becoming more and more popular in precision medicine, not limited to CF.^51^^,^^52^ However, considering CF, off-label prescription of the currently available CFTR modulators is possible in some but not all countries, and a lack of indication may allow third-party payers (e.g., health insurance companies and local health authority funders) to deny access regardless of the efficacy proven by tests *in vitro*. In these specific circumstances (ultra-rare variants), running a classic clinical trial is clearly impossible.^51^ Based on the reported case and available literature, it seems that a revision of the off-label drug prescription policy in some contexts, always based on objective data provided by solid scientific references, should be a priority for regulation authorities, as science runs faster than regulations. The scientific evidence, stemming from an increasing number of cases studied with this approach (testing patient-derived cells), is expected to identify the most efficient and cost-effective methodology, including the cell model and the type of assay better suited to predict drug efficacy, thus providing solid arguments for policymakers. It is of relevance in our view to consider that cellular models can be properly stored for later use and can be utilized to refine the therapy for individuals, allowing the testing of new drugs when made available. Considering the increasingly high number of tailored approaches and drugs that are being evaluated by numerous pharmaceutical companies, this would offer an unprecedented opportunity to rapidly test the potential efficacy of a new drug/approach without need of resampling, following the evolution of the field in a clinically timely manner for the benefits of the patients.

Herein, we showed an overall concordance of the results obtained in 3D and 2D assays, including protein expression analysis, in both intestinal and nasal organoids suggesting that both models are appropriate to evaluate the response to CFTR modulators with no gross differences that might jeopardize the conclusions. These findings support the potential efficacy of all the drugs tested, which was possible to confirm *in vivo* only for Kalydeco, being this treatment approved by local authorities for this patient. In our view, it is reasonable to propose that any data produced in established *in vitro* cellular models should justify a short period of treatment with additional formulations monitored by CFTR function assays performed *in vivo*, which might not be limited to the sole Gibson and Cooke sweat test. A final decision on the treatment should follow, based on the analysis of the functional and clinical data collected.

### Limitations of the study

Being this an exceedingly rare case, our data suggest that in similar cases the response to a CFTR modulator in any of the primary cell samples here evaluated might correctly guide the clinician to a specific treatment. Obviously, this approach cannot be generalized without the collection of larger series of cases, necessary to evaluate the robustness of the models here utilized.

## STAR★Methods

### Key resources table

**Table:** 

REAGENT or RESOURCE	SOURCE	IDENTIFIER
**Antibodies**		

α-CFTR monoclonal antibodies 450	CFTR Antibody Distribution Program, Cystic Fibrosis Foundation, UNC-Chapel Hill	N/A
α-CFTR monoclonal antibodies 570	CFTR Antibody Distribution Program, Cystic Fibrosis Foundation, UNC-Chapel Hill	N/A
α-CFTR monoclonal antibodies 596	CFTR Antibody Distribution Program, Cystic Fibrosis Foundation, UNC-Chapel Hill	N/A
horseradish peroxidase-conjugated secondary antibody α-mouse	Cell Signalling	Cat# 7076; RRID: AB_330924
mouse monoclonal β-actin clone AC-15	Sigma-Aldrich	Cat# A5441; RRID: AB_476744
α-beta-actin antibody	Cell Signalling	Cat# 4970; RRID: AB_2223172

**Chemicals, peptides, and recombinant proteins**		

Dimethyl sulfoxide (DMSO)	Sigma Aldrich	Cat#D2650
Matrigel	Corning	Cat#354262
0.25% Trypsin	Corning	Cat#25-050-Cl
Advanced Dulbecco’s Modified Eagles Medium with Nutrient Mixture F-12 Hams (Ad-DF) 500 mL	Gibco	Cat#12634010
Matrigel Growth Factor-Reduced	Corning	Cat#354230
RPMI-1640	Thermo Fisher Scientific	Cat#11875093
Glutamax	Thermo Fisher Scientific: Invitrogen	Cat#35050
HEPES	Corning	Cat#25060-Cl
Penicillin/streptomycin	Thermo Fisher Scientific: Invitrogen	Cat#15140-122
Gentamicin	Life Technologies: Gibco	Cat#15710-049
EDTA	Sigma	Cat#03690
VX-770	Selleck Chemicals LLC	Cat#S1144
VX-809	Selleck Chemicals LLC	Cat#S1565
VX-661	Selleck Chemicals LLC	Cat#S7059
VX-445	Med Chem Express	Cat#HY-111772
Y-27632 Dihydrochloride (ROCKi)	Merk Life Science	Cat#Y0503
CHIR-99021	Merk Life Science	Cat#SML1046
PneumaCult–ALI Medium	STEMCELL Technologies	Cat#05001
Vancomycin	Sigma Aldrich	Cat#861987
Forskolin	Sigma	Cat#F3917
Collagen, type IV, from human placenta	Sigma	Cat#234154
Primocin (50 mg/ mL)	Invivogen	Cat#ant-pm-1
Cell recovery solution	Corning	Cat#354253
Westar Supernova ECL substrate detection	Cyanagen	Cat#XLS3
NuPAGE™ Sample Reducing Agent	Invitrogen	Cat#NP0009
NuPAGE Tris-Acetate	Invitrogen	Cat#EA0375BOX
pcDNA5/FRT expression vector	Thermo Fisher Scientific	Cat#V601020
Lipofectamine™ 3000 Transfection Reagent	Thermo Fisher Scientific	Cat#L3000150
RIPA Lysis and Extraction Buffer	Thermo Fisher Scientific	Cat#89901
Halt protease inhibitor mix	Thermo Fisher Scientific	Cat#78430
Roche cOmplete, Mini Protease Inhibitor Cocktail	Roche	Cat# 11836153001

**Experimental models: Cell lines**		

CFBE41o- Human CF Bronchial Epithelial Cell Line	ATCC	N/A
Fischer rat thyroid gland (FRT) cells	ATCC	N/A
HEK293	ATCC	N/A
Human colon organoids genotype W57G/A234D	General Pathology Lab, University of Verona	N/A
CRC-derived nasal organoid genotype W57G/A234D	Department of Oncology and Molecular Medicine, Istituto Superiore di Sanità	N/A
CALU3 lung adenocarcinoma	ATCC	N/A
Human colon organoids genotype F508del/F508del	General Pathology Lab, University of Verona	N/A
Human colon organoids non-CF	General Pathology Lab, University of Verona	N/A

**Software and algorithms**		

Graphpad Prism	Graphpad	https://www.graphpad.com/scientific-software/prism/
Microsoft Excel	Microsoft Corporation	https://office.microsoft.com/excel
ImageJ	National Institutes of Health	https://imagej.nih.gov/ij/
PowerPoint software	Microsoft Corporation	https://office.microsoft.com/powerpoint
Labchart v8	AD Instruments	https://www.adinstruments.com/products/labchart
Image Lab™ Software	BioRad	N/A

**Other**		

EVOS Cell Imaging System, with 4x objective, incorporated incubator (allowing controlling temperature and CO_2_)	Thermo Fisher Scientific	N/A
6.5 mm Transwell® inserts	Costar	Cat#3470
Olympus CKK31 inverted microscope	Olympus	N/A
EVOM2 Epithelial Voltohmmeter	World Precision Instruments	N/A
EVC4000 multi-channel voltage/current clamp	World Precision Instruments	N/A
Easymount Ussing Chamber	Physiologic Instruments	Cat#P2300
EasyMount Ussing Chamber Sliders	Physiologic Instruments	Cat#P2302T
PowerLab	AD Instruments	N/A
12 mm Transwell® inserts	Corning	Cat#3460
ChemiDoc™ XRS+	BioRad	N/A
Time-lapse imaging station	Olympus	N/A
4–15% Criterion™ TGX™ Precast Midi Protein Gel	BioRad	Cat#5671083

### Resource availability

#### Lead contact

Further information and requests for resources and reagents should be directed to and will be fulfilled by the lead contact, Claudio Sorio (claudio.sorio@univr.it).

#### Materials availability

This study did not generate new unique reagents.

### Experimental model and study participant details

#### Institutional Review Board Statement

This study was conducted according to the guidelines of the Declaration of Helsinki, and approved by the Institutional Review Board CRCFC-CFTR028 on 12 June 2019, and the patient signed informed consent.

#### Clinical data

The patient was a 37-year-old female of Italian origin, with abnormal sweat Cl^–^ (112 mmol/L), pancreatic sufficiency (PS), lung infection (*Pseudomonas aeruginosa*), impaired lung function (forced expiratory volume in the first second: FEV1): 64% of the predicted value), bilateral bronchiectasis, kidney stones, recurrent sinusitis. She was CF-diagnosed at three years of age by symptoms. Both her intestinal current measurement (ICM), nasal potential difference (NPD), according to the European CF Society SOPs, were consistent with the diagnosis of CF. Defective CFTR function was assessed in this patient also by the optical beta-adrenergic sweat test, performed twice with overlapping results featuring the absence of sweat droplets, following induction with beta-adrenergic stimuli.^53^ The allelic segregation in this patient was established following analysis of her mother and sister, while the father was not alive. CFTR complex alleles after detection of the W57G/A234D genotype by denaturing high-performance liquid chromatography (DHPLC) were excluded in this patient according to the results of further genetic analyses performed by next-generation sequencing (NGS), multiplex ligation probe amplification (MLPA), and RNA analysis of cells obtained by nasal brushing. The patient was treated with ivacaftor off-label, and *in vivo* outcomes such as lung function by spirometry (FEV1), sweat chloride by the Gibson and Cooke sweat test (GCST) and, CFTR-dependent sweating monitored by imaging^42^ were acquired up to 27 weeks.

#### Participants’ biospecimen collection

Rectal biopsies and nasal brushings were collected from CF participants with W57G/234D (n = 1) and F508del/F508del (n = 1), as well as a wild-type CFTR (WT-CFTR) control participant (n = 1). The local ethical committee approved this study (CRCFC-CFTR028) and written informed consent was obtained from all participating subjects.

#### Crypts isolation and organoid culture from nasal and rectal specimens

Briefly, human rectal biopsies collected with colon forceps were immediately stored at 4°C in surgical medium (RPMI-1640, glutamax 1X, HEPES 10 mM, penicillin/streptomycin 1%, 10 μg/mL of gentamicin, and 20 μg/mL of ciprofloxacin). Biopsies were then washed with a cold PBS solution and incubated in 10 mM EDTA for 90–120 min at 4°C. After washing, the isolated crypts were mixed with 50% Matrigel (growth factor-reduced, phenol red-free, Corning, New York, USA) and sown in 30 μl per well (with approximately 20–30 crypts/10 μL of Matrigel/droplet) in pre-warmed 24-well plates. After Matrigel polymerization for 15–30 min at 37°C, the plated crypts were covered with pre-warmed complete medium supplemented with 10 μM of Rho inhibitor (Y27623) and 10 μM of Chir (CHIR-99021) (both from Merk Life Science, Milano, Italy). Additional antibiotics (gentamycin and vancomycin, both at 50 μg/mL; Sigma) were used during the first week of culture. The medium was refreshed every other day, and outgrowing crypts/organoids were expanded 1:3–1:5 times every 7–10 days.

Nasal epithelial cells were obtained through cytology brushing. For CRC-derived nasal organoid generation we followed established protocols.^25^ Briefly, conditionally reprogrammed nasal epithelial stem cells (CRC cells) expanded *in vitro* based on previously published protocols,^25^ were suspended at 30000 cells/100 Micro μL in Matrigel Growth Factor-Reduced (Corning), carefully pipetting to generate a single-cell suspension. One hundred μL aliquots were seeded into 24 well plates, creating a “drop” of matrigel and incubated at 37°C and 5% CO_2_ for 30 min, to allow matrigel setting. CRC growth medium (0.5 ml) was overlayed to cover the matrigel drop and replaced after 3-4 days with fresh medium. Seven days after plating CRC medium was replaced with PneumaCult–ALI Medium (STEMCELL Technologies, Vancouver, Canada) to induce organoid maturation. Medium was replaced every other day with fresh medium for a total of 21 days.

### Method details

#### CFTR genotype: Description of the DNA variants analyzed in this study

The p.Trp57Gly mutation (legacy name: W57G is a very rare variant (0,007% of allele frequency) caused by a T > G transversion at cDNA nucleotide position c.169 located in exon 3, resulting in a tryptophan to glycine amino acid substitution at codon 57 located in the lasso motif. Mutations in this region are described to cause intracellular retention or abnormal gating.^54^ This variant was reported in the Cystic Fibrosis Mutation Database by M Ferrari in a 26-year-old Italian female diagnosed at 22 years of age with pancreas sufficiency (PS), sweat chloride of 101 mmol/L, and severe lung disease (FEV1 = 35%), who had a lung transplant when she was 30 years old. The R352Q variant was present on the other allele. The W57G variant causes CF when combined with another CF-causing variant in trans. To date, 10 patients with this variant are reported in the CFTR2 database. This variant is reported to have a very small residual function (about 1% of WT CFTR)^44^ and respond poorly to ivacaftor and lumacaftor alone and, to a slightly higher extent, to this combination, suggesting a marginal additive effect.^43^^,^^44^ Furthermore, no processing of mature band (C band) nor functional response was reported following treatment with VX661 and VX661+445^45^ and Figure 7C.

The p.Ala234Asp mutation (legacy name: A234D, c.701C>A) is caused by a C > A transversion at cDNA nucleotide position c.701 located in exon 6, resulting in an alanine to aspartate amino acid substitution at codon 234. This variant is poorly described: we could only retrieve a report from Porcaro et al. describing this variant in a group of patients from the Lombardy region in Italy, thus excluding it is a *de novo* mutation.^55^ This variant appears to present a residual function (17% of WT) and to be responsive to VX770 (56% of WT).^46^ This variant is still not reported in the CFTR2 database and is classified as probably pathogenic (VUS5, according to MAPP and SWIFT) in the Inserm database (access on July 26^th^, 2023) but it is indicated as responsive to CFTR modulators (https://www.vertextreatments.com). Recently an approach that utilize a population data analysis Bayesian prevalence ratio (BayPR) score was developed.^56^ When applied to this variant it provides a 99% probability of being disease-causing.

The CFTR genotype in this patient was detected by DHPLC, and then confirmed by NGS and MLPA; no evidence of aberrant splicing was obtained from analysis of RNA in nasal brushing. Segregation analysis was performed by testing the mother and the sister of this patient; since both are healthy carriers of the CFTR variant A234D, the variants A234D and W57G in the patient are in trans.

#### Molecular modeling of open and closed conformations of CFTR and in silico analysis of the effects of the W57G and A234D variants on protein stability

Molecular modeling analyses were performed within the environment of the chemical simulation software Maestro/Bioluminate (Schroedinger, New York, USA). The electron cryomicroscopy (cryo-EM) structure of the ATP-free, dephosphorylated form of CFTR (PDB entry: 5UAK1, resolution: 3.87 Å) was used as a reference for the closed channel conformation. The structure of CFTR in its open conformation was modeled based on the ATP-bound state of CFTR in complex with VX770 (ivacaftor), solved also by cryo-EM (PDB entry: 6O1V2, resolution: 3.3 Å). Protein structures were prepared according to the pipeline of the Bioluminate Protein Preparation tool. Briefly, the following steps were taken: (i) assignment of bond orders using the Chemical Components Dictionary database (www.pdb.org); (ii) addition of H atoms; (iii) creation of missing side chain and loops using Prime; (iv) selection of the most probable rotamer and deletion of water molecules closer than 3.5 Å to other water molecules. The resulting structures were refined by steps including sampling of water orientation and prediction of the protonation states of ionizable residues at pH 7.5 by PROPKA prior to H-bond assignment and optimization. Finally, each protein structure was minimized using the OPLS_2005 forcefield (Schroedinger, New York, USA) until the root mean square displacement of the heavy atoms reached 0.3 Å.

The Protein Surface Analysis tool was used to analyze the solvent-accessible surface areas of each residue, as well as the distribution of hydrophobic and electrostatic surface patches. The Residue Scanning tool was used to generate, *in silico*, the W57G and A234D mutations. All residues located less than 5 Å from the site of the mutation were energy-minimized (both backbone and sidechain, when appropriate). The Molecular Mechanics/Generalized Born and Surface Area Continuum Solvation (MM/GBSA) method was used to predict the effects of residue mutation on protein stability. A case-specific thermodynamic cycle was used to determine the relative changes in free energy compared to the WT. It should be noted that the free energies computed by this method are based on the MM force field and exclude any explicit contributions from conformational changes. The obtained values (ΔΔGapp, expressed in kcal/mol) should therefore be taken as “apparent” values and considered as approximate indexes useful in comparisons, rather than rigorous thermodynamic quantities. Molecular structure figures (Figures 1 and 2) were prepared using Maestro/Bioluminate.

#### Stead-state total organoid area (SOA) assay and forskolin-induced swelling (FIS) assay (intestinal specimens)

Rectal organoids (OGs) from a 7- to 10-day-old culture were disrupted mechanically and seeded in 96-well plate in 5μl of 50% Matrigel (Corning) containing 20-30 organoids and immersed in 50μl of culture medium with one of the following treatments: DMSO (0.1%), 3μM VX809 (Selleck Chemicals LLC, Monmouth Junction, USA), 3μM VX661 (Selleck Chemicals LLC, Houston, USA) and 2μM VX445 (Med Chem Express), or their combination. After 2 hours of seeding and after 20-24 hours of treatment, treated organoids were analyzed by microscopy for taking one picture at t=0hpt (0 hour post treatment) and another picture at t=24hpt (24 hours post treatment) (EVOS Cell Imaging System, Thermo Fisher Scientific) with 4x objective, at 37°C and 5% CO_2_). The total area (xy plane) of the majority of organoids in a well was analyzed, and the SOA of the selected organoids was calculated manually using Image J and GraphPad Prism version 7 (GraphPad Software, San Diego, California, USA). The SOA was expressed as area under the curve (AUC t = 24h; baseline, 100%). Cell debris and nonviable structures were excluded from the analysis. The same group of treated organoids were used for the subsequent FIS assay.

For performing the FIS experiments, at the day of the assay, we acquired pictures of treated organoids at 10 min intervals for a total acquisition of 1 h using EVOS Cell Imaging System (Thermo Fisher Scientific) with a 4x objective, at 37°C and 5% CO_2_. For CFTR potentiation, 3 μM of VX770 (Selleck Chemicals LLC) was added simultaneously with forskolin. The organoid swelling was measured (xy plane) and related to t = 0 (prior the addition of forskolin) and to forskolin treatment (FSK 0.02, 0.128, 0.8, and 5 μM) as follows: starting from the image acquired at time t = 0, organoids were numbered progressively, using PowerPoint software, until selected organoids were numbered, creating a mask. The subsequent overlapping of the mask to images ensured the evaluation of the same set of organoids over time. Then, the circumference of each numbered organoid was measured using ImageJ software^57^ and a freehand selections tool, and the corresponding area was automatically calculated in pixels by the software. Normalized data are expressed as total area under the curve (AUC, t = 60 min; baseline, 100%) calculated using GraphPad Prism version 7 (GraphPad Software, San Diego, California, USA). A Kruskal–Wallis test for multiple comparisons was used to calculate statistical differences, and *p* < 0.05 was considered statistically significant.

#### Two-dimensional monolayer culture and Micro-Ussing chamber recordings (intestinal specimens)

Transwell®, 6.5 mm with 0.4 μm Pore Polyester Membrane Insert Filters (Ref. 3470, Corning) compatible with a 24-Well Transwell HTS plate (Ref. 3378, Corning) were previously coated overnight with collagen, type IV, from human placenta (10 μg/cm^2^) (234154, Sigma) diluted in saline phosphate buffer and incubated at 37°C for at least 2 h. For culture of epithelial monolayers, seven-day-old, extracellular matrix-embedded, intestinal organoids were suspended in advanced DMEM F12 (4°C; Gibco) and washed by centrifugation (5 min, 1,500xg) to remove the matrix. Intestinal organoids were dissociated by brief (45 sec, 37°C) incubation in trypsin (0.25%) solution (Corning), followed by mechanical disruption through a 200 μL pipette tip (Greiner). Such step was repeated for the times necessary to obtain a single cells suspension. Then, 250,000 cells were seeded for each insert (filter), with a 100 μL organoid culture medium added on top and 600 μL of organoid culture was added on the bottom side of the filters. Organoid monolayers were cultured in a 5% CO_2_ atmosphere at 37°C. The culture medium was supplemented with Y27632-Rho and CHIR-99021 (both from Sigma-Aldrich) during the first two days after seeding. The organoid culture medium (without supplement) was changed every other day. After a minimum of 7–10 days of cell culture, the formation of organoid monolayers was monitored by morphologic observation using an Olympus CKK31 inverted microscope (Olympus, Japan) and measurement of transepithelial electrical resistance (TEER) using EVOM2 Epithelial Voltohmmeter (World Precision Instruments) before refreshing the medium. The unit area resistance (Ω⋅cm^2^) is obtained by multiplying the meter readings by the effective surface area of the Transwell inserts (0.33 cm^2^) to calculate the unit area of resistance (Ω⋅cm^2^).

Monolayers with transepithelial electrical resistance (TEER) values around 400 Ω.cm^2^ were considered ready for the transepithelial short-circuit current (Isc) measurement and drug treatments. Filters were mounted on a slider (P2302T; 0.33 cm^2^ aperture) in an Ussing chamber (P2300, PI) and Isc was measured with a EVC4000 multi-channel voltage/current clamp (WPI, World Precision Instruments). Recordings were performed filling the two half chambers with a symmetric apical and basolateral Meyler saline buffer solution (Hepes 10 mM; Na_2_HPO_4_ 0.3 mM; NaH_2_PO_4_ 0.4 mM; MgCl_2_ 1.0 mM; CaCl_2_ 1.3 mM; KCl 4.7 mM; NaCl 128 mM; NaHCO_3_ 20.2 mM; D-Glucose 10 mM, pH 7.4, and osmolarity 300 mOsm). The buffer was maintained at 37°C and carbogen (95% O_2_, 5% CO_2_) was constantly gassed. After stabilization of the transepithelial current, baseline Isc values were recorded. Subsequently, the ENaC blocker Amiloride (100 μM), the cAMP agonist forskolin (FSK; up to 10 μM), the CFTR potentiator VX770 (1 μM), and, at the end of the test, the CFTR inhibitor PPQ-102 (20 μM, Tocris) were added both to the apical and the basolateral sides, but amiloride that was added only at apical side. When indicated, 2D monolayers were pre-incubated for 24 h with the CFTR correctors VX809 or VX661 (3 μM, Selleck Chemicals LLC) with or without the CFTR type III corrector VX445 (2 μM, Med Chem Express). The tracings were recorded with PowerLab (8/35, AD Instruments) and the data were analyzed with Labchart v8 software (AD Instruments). We calculated the Isc changes (ΔIsc) by taking the difference in the Isc recorded after adding the secretagogues and or inhibitor listed above. Statistical analysis was performed with Prism7 software, and the Kruskal–Wallis test for multiple comparisons was performed.

#### Western blot analysis (intestinal specimens)

Untreated 3D organoid or those treated with CFTR correctors were disrupted with advanced DMEM/F12 supplemented with 1% glutamax, 10mM Hepes, 0.2% primocin, and 1% penicillin/streptomycin. The Matrigel matrix was removed from the three-dimensional structures with cell recovery solution (Corning) following the manufacturer’s instructions. The pellet was lysed with 50 μL of RIPA/EDTA/DTT/vanadate lysis buffer (50 mM Tris, pH 7.5, 15 0mM NaCl, 1% Triton X-100, 1% sodium deoxycholate, 0.1% SDS, 1 mM EDTA, 1 mM DTT, and 1 mM sodium orthovanadate) with 1x protease inhibitor cocktail (Roche), and then disrupted by vigorous pipetting with a P200 tip or by vortexing. For cells grown on 2D filters, they were washed once with PBS and then lysate in 35 μL of RIPA buffer as above. After 30 min at 4°C under agitation, the lysate was spun at 13,000x rpm at 4°C (maximum radius of 8.5 cm; Biofuge Fresco, Heraeus, Hanau, Germany) and the supernatant was collected and subjected to protein concentration analysis (Bradford). Then, 30–60 μg of total protein was further solubilized in 4x Laemmli sample buffer (4% SDS, 5% β-mercaptoethanol, 20% glycerol, 0.0025% bromophenol blue, 0.16 M Tris-HCl, and pH 6.8) with 125 mM DTT and incubated at 37°C for 20 min. Proteins were resolved by SDS–polyacrylamide gel electrophoresis using 7.5% acrylamide gels. Proteins were wet-transferred from gel to a polyvinylidene difluoride (PVDF) membrane by electrophoresis with a transfer system (Bio-Rad) at 100 V (constant) for 60 min. The membrane was blocked with 5% non-fat dry milk/Tris-buffered saline (TBS)–Tween (0.3%) at 25°C for 1 h, then incubated with α-CFTR monoclonal antibodies (mAb) 450, 570, and 596 (CFTR Antibody Distribution Program, Cystic Fibrosis Foundation, UNC-Chapel Hill) (1:1000 dilution each) at 4°C overnight. The membrane was washed four times for 10 min each with TBS–Tween (0.3%) and then incubated with horseradish peroxidase-conjugated secondary antibody α-mouse at 1:12,000 (Cell Signaling, 7076) at 25°C for 1 h. The membrane was again washed four times for 10 min each with TBS–Tween (0.3%) and enhanced chemiluminescence development by Westar Supernova ECL substrate detection (Cyanagen). The relative chemiluminescence intensity of the target protein was normalized to the chemiluminescence intensity of β-actin detected by α-beta-actin antibody (1:1000) (Cell Signaling, 4970). ImageJ software (version 1.8.0112, National Institute of Health, Bethesda, 144 MD, USA) was used to analyze the band densities, and the results were confirmed by a minimum of three independent experiments.

For all experiments, protein lysates from non-CF (healthy control: HC), CALU3 lung adenocarcinoma cell line (from ATCC, cultured in RPMI medium with 10% serum) and F508del/F508del-derived intestinal organoids were used where indicated as appropriate controls for CFTR expression in the immunoblot.

#### Differentiation of conditionally reprogrammed cells (CRC) under Air-liquid interface (ALI) culture conditions, drug treatment, immunoblot Ussing chamber analysis and FIS assay (nasal brushing-derived specimens)

CRC cultures were performed as previously described.^25^ To induce differentiation, 1.1 x 10^5^ CRC cells were plated in 12 mm Transwell® plates with a 0.4 μm Pore Polyester Membrane Insert (Corning) and cultured in CRC complete medium in both basal and apical chambers until confluence was reached (seven days) with medium replacement after 3 days culture. Afterward, CRC medium was replaced with PneumaCult–ALI Medium (STEMCELL Technologies, Vancouver, Canada) in the basal chamber, leaving the apical chamber empty for 28 days, with medium replacement every other day for 28 days. After differentiation of the CRC under ALI culture conditions (as described above), the cells were left untreated or treated with drugs or drug combinations (in triplicate) for two days (in the basolateral chamber). The cells were exposed to the following drugs in the last 48 h of differentiation: VX809 (5 μM), VX661 (10 μM), VX445 (3 μM), or combinations thereof before cell lysis. To obtain cell lysates, ALI-cultured cells were washed twice with PBS and detached from the transwells through trypsin incubation, for 20 minutes at 37°C. After further washing, cell pellets were incubated on ice for 30 minutes in the following lysis buffer: 1%NP40, 0,15 M NaCl, 50 mM Tris pH 7,4, protease and phosphatase inhibitors (Sigma). After centrifugation for 10 minutes, 4°C at 13000 RPM (18000g with the Eppendorf 5417R refrigerated microfuge equipped with a 45-30-11 rotor) to remove insoluble cell debris, the supernatant was used for protein determination.

For the immunoblotting studies, 20 μg of total lysate proteins from each sample were added with NuPAGE™ Sample Reducing Agent, loaded (without boiling) and resolved on 3%–8% polyacrylamide gel electrophoresis NuPAGE Tris-Acetate (Invitrogen, Carlsbad, CA, USA) and transferred to nitrocellulose membranes. The following primary antibodies were used: α-CFTR monoclonal antibodies (mAb) 596 (CFTR Antibody Distribution Program, Cystic Fibrosis Foundation, UNC-Chapel Hill, dilution 1:1000) and mouse monoclonal β-actin (Sigma-Aldrich, clone AC-15, dilution 1:10000) antibodies. Peroxidase-conjugated secondary antibodies were purchased from Amersham™ and used 1:4000. Quantification of the immunoblot band intensity was performed through Image lab software (Chemidoc XRS+, Biorad). To quantify CFTR maturation, the relative amount of CFTR band-C protein was normalized to β-actin, measured in the identical protein sample, and these levels were used for subsequent calculations.

Ussing chamber analysis was performed as previously described.^58^

For CRC-derived nasal organoid FIS assays, organoids were pre-treated with VX809, VX661, VX445 or their combinations (all drugs purchased from Selleck Chemicals), for 48 hours, at the same doses as described for immunoblot analyses. Spheroid images were captured (10X magnification) using Time-lapse imaging station (Olympus, Tokyo, Japan), at time 0 and after 2 days of subsequent stimulation with 5μM VX770 (Ivacaftor) or 5μM VX770 + different concentrations of Forskolin (from 0.128 to 10μM; Selleck Chemicals), to monitor and assess spheroid swelling (n = 10 spheroids per condition for all experiments described were analyzed). Images were analyzed by manually delineating the area of each spheroid before and after treatments using ImageJ software. Spheroid area data were imported into Microsoft Excel and percent change (post stimulation vs basal) was calculated for each individual spheroid. Data were reported as area under the curve (AUC).

#### Expression of A234D variant in FRT and CFBE cells

cDNA encoding A234D CFTR was cloned into the pcDNA5/FRT expression vector (Thermo) between NotI and XhoI restriction sites as previously described.^43^ For immuno blots, HEK293 (ATCC) cells in 10 cm plate were transfected with 8μg of A234D expression plasmid using Lipofectamine 3000 (Thermo) according to manufacturer’s protocol. The transfected cells were equally divided and seeded into five wells of a 6-well plate. The indicated compounds were added into each well the next day and incubated for 24 hours before harvesting cells. Cell pellets collected from each well were lysed with RIPA lysis buffer (Thermo) containing Halt protease inhibitor mix (Thermo). Fifty μg of total lysate was resolved for each sample using 4-15% Criterion gel (Biorad) and blotted onto nitrocellulose membrane. CFTR bands were probed with α-CFTR monoclonal antibodies (mAb) 596 (CFTR Antibody Distribution Program, Cystic Fibrosis Foundation, UNC-Chapel Hill) and β-actin was probed as a loading control using monoclonal antibody (Sigma, USA). Short-circuit current (Isc) measurements were done by transfecting FRT cells on filters as previously described.^59^ A234D variant was also expressed in CFBE that were transfected and analyzed exactly as previously described.^43^^,^^44^

#### Optical ratiometric measurement of CFTR-dependent sweat production

An intradermal microinjection (100 μL) of methacholine (M)-stimulated CFTR-independent sweating in the M phase, and beta-adrenergic agonists induced CFTR-dependent sweat secretion in the C phase following an intradermal microinjection of aminophylline and isoprenaline with atropine were used as a cholinergic inhibitor. Individual sweat glands were totaled by visually counting single spherical sweat droplets (“bubbles”) in a water-saturated oil layer, including dispersed, water-soluble blue dye particles (350 μL in a 1 cm diameter well). Single glands were mapped (ImageJ software) on pictures acquired 10 or 30 min after microinjections of methacholine or beta-adrenergic cocktail (C), respectively, and the sweat volume secreted per minute and the C/M ratio were calculated exactly as previously described.^42^

### Quantification and statistical analysis

Data were analyzed using Ordinary one-way or two-way ANOVA with Kruskal–Wallis’s multiple comparison test in Prism 7 (GraphPad). Statistical significance was considered p < 0.05, and differentiation was made between p < 0.05, p < 0.009, p < 0.005, p < 0.002, p < 0.0002, p < 0.0001 and p < 0.00001.

Statistical analysis was performed with Prism7 software, and the test for multiple comparisons was used to calculate statistical differences, and *p* < 0.05 was considered statistically significant.

## Data Availability

• Upon request, the lead contact will share the original data reported in this article. • This paper does not report original code. • Any additional information required to reanalyze the data reported in this paper is available from the lead contact upon request.

## Supplemental information

Video S1. Recording of cilia beating in nasal organoids, related to Figure 4
