# Supplementary material for: In silico analysis and theratyping of an ultra-rare CFTR genotype (W57G/A234D) in primary human rectal and nasal epithelial cells
Source: iScience. 2023 Oct 12;26(11):108180. doi: 10.1016/j.isci.2023.108180 (PMC10660498; doi:10.1016/j.isci.2023.108180)
Supplement: Document S1. Figures S1–S4 and Table S1 [file mmc1.pdf]

iScience, Volume *26*

Supplemental information

In silico analysis and theratyping of an ultra-rare CFTR genotype (W57G/A234D) in primary human rectal and nasal epithelial cells

Karina Kleinfelder, Virginia Lotti, Adriana Eramo, Felice Amato, Stefania Lo Cicero, Germana Castelli, Francesca Spadaro, Alessia Farinazzo, Daniele Dell'Orco, Sara Preato, Jessica Conti, Luca Rodella, Francesco Tomba, Angelo Cerofolini, Elena Baldisseri, Marina Bertini, Sonia Volpi, Valeria Rachela Villella, Speranza Esposito, Immacolata Zollo, Giuseppe Castaldo, Carlo Laudanna, Eric J. Sorsher, Jeong Hong, Disha Joshi, Garry Cutting, Marco Lucarelli, Paola Melotti, and Claudio Sorio

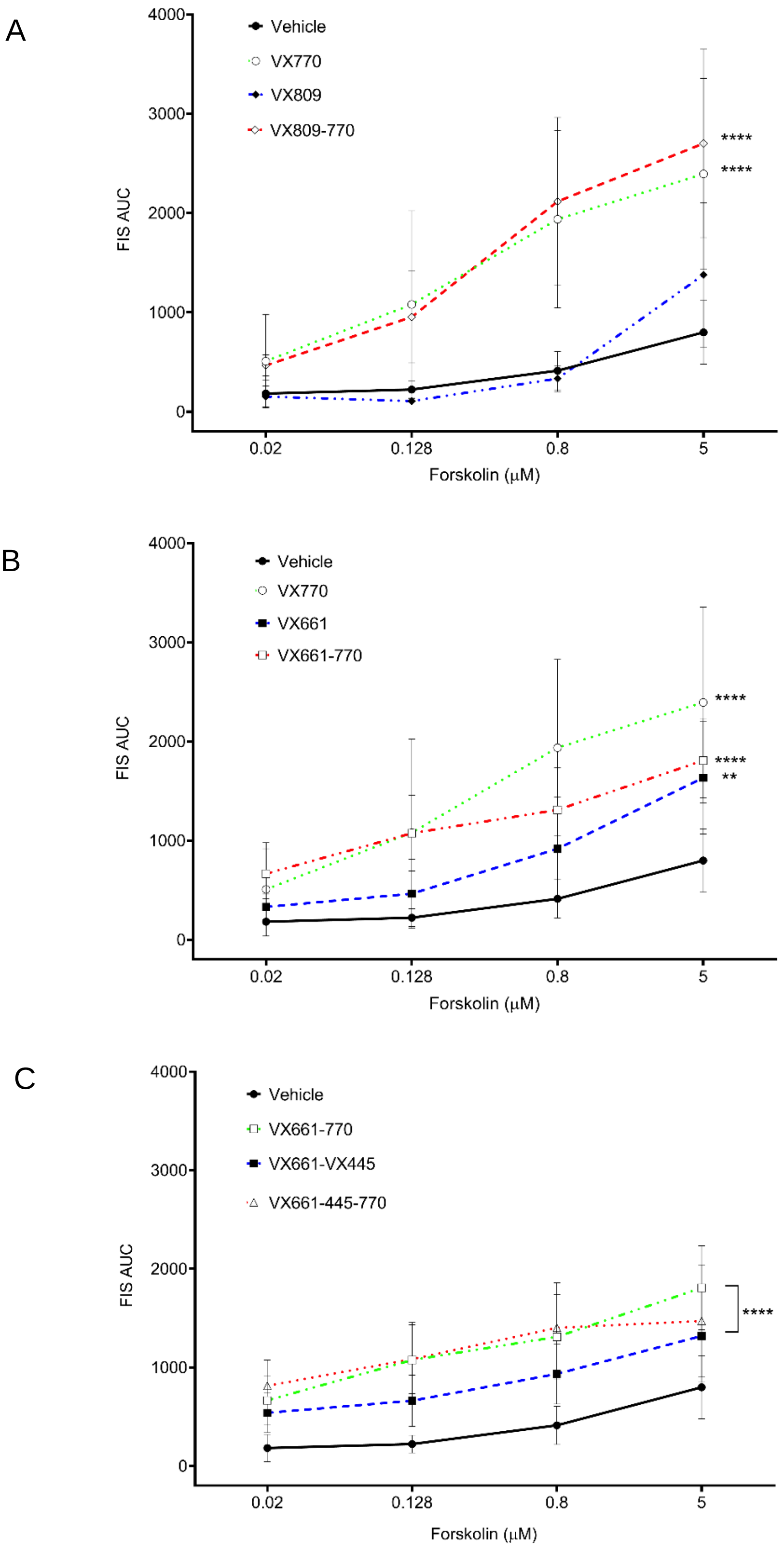

Figure S1. Functional characterization of W57G/A234D colonoids by the FIS assay, related to Figure 3. Colonoids were pre-incubated for 24 h with the vehicle (DMSO 0.1% v/v), 3μM VX809 (A), 3μM VX661 (B), and 3μM VX661 + 2μM VX445 (C) at the indicated doses. At the time of the assay, colonoids were stimulated with various concentrations of forskolin (from 0.02 to 5 μM). VX770 (3 μM) was added when indicated. ** p=0.005, ***, p=0.0006, **** p<0.0001 two way ANOVA.

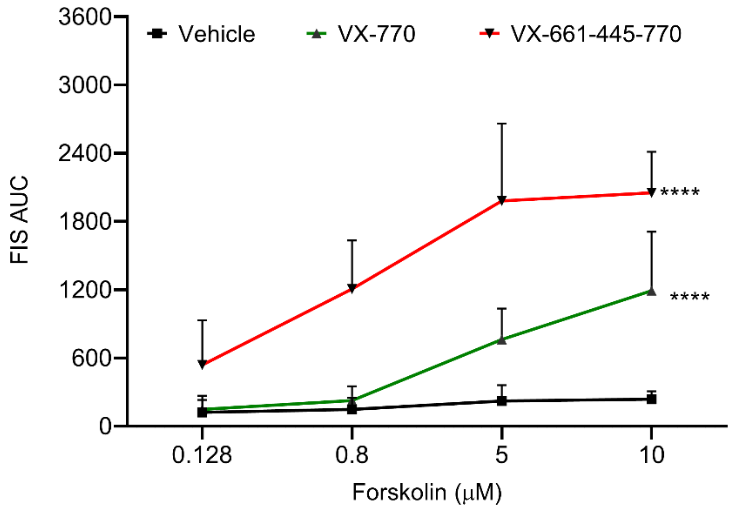

Figure S2. W57G/A234D nasal organoids forskolin (fsk) dose response in FIS assay, related to Figure 4. Area under the curve (AUC) of nasal organoids pre-incubated for 48 h with the vehicle (DMSO 0.1% v/v) and VX661 + VX445, responding to fsk dose (from 0.128 to 10 μM). VX770 was added for additional 48 hours, together with fsk, when indicated. **** $p<0.0001$ two way ANOVA.

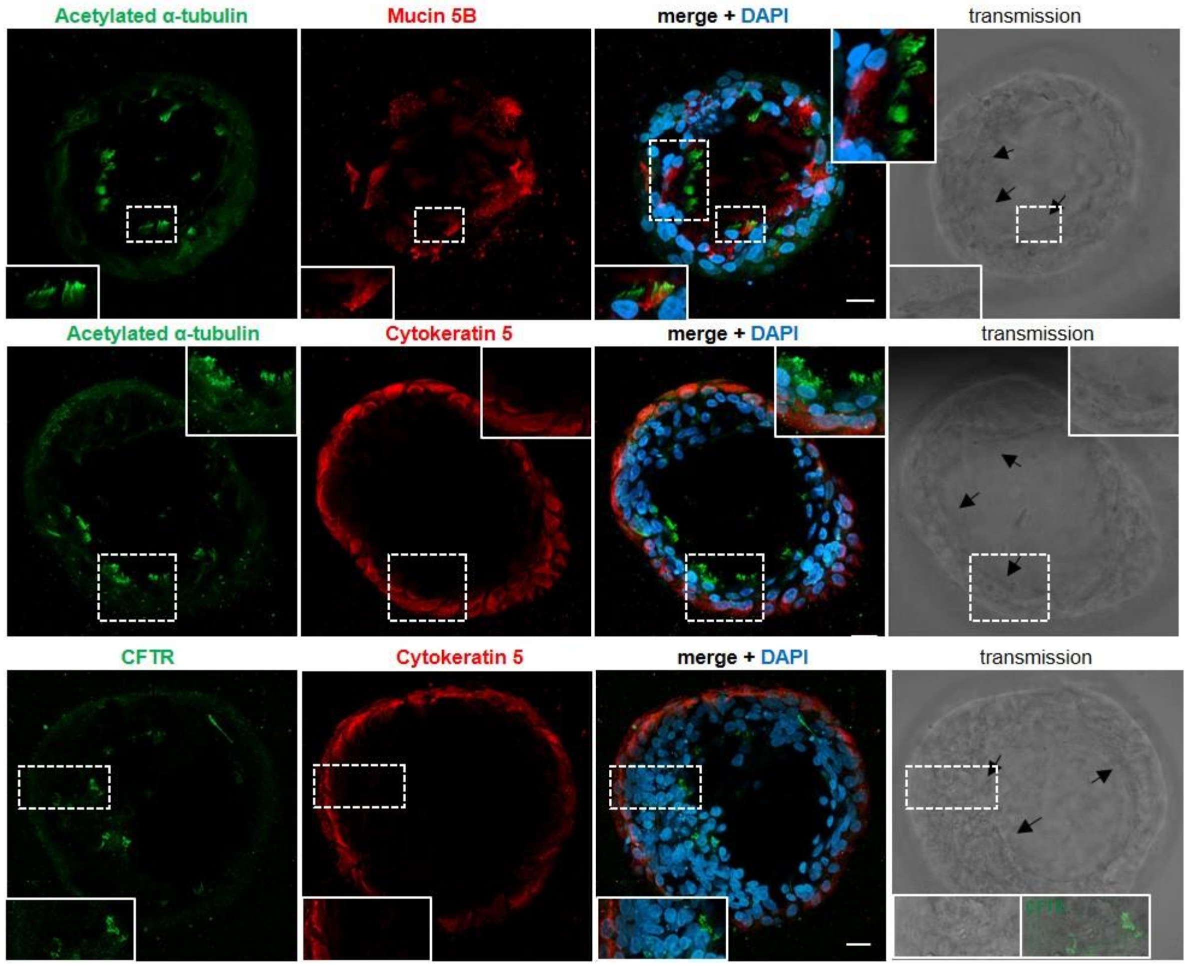

Figure S3. CLSM examinations (orthogonal projections of central optical sections) of intact organoids in matrigel generated from W57G/A234D patient cells, related to Figure 4. Organoids were fixed, permeabilized and stained for acetylated α-tubulin (green) or CFTR (green) in combination with Mucin 5B (red) or cytokeratin-5 (red). Nuclei are stained in blue (DAPI). Separate channels and merged images are shown. Insets represent higher magnification images of selected

ROI (region of interest). On the right, transmission light image with arrows indicating cilia in the luminal side of the organoids. Scale bars, 20 µm.

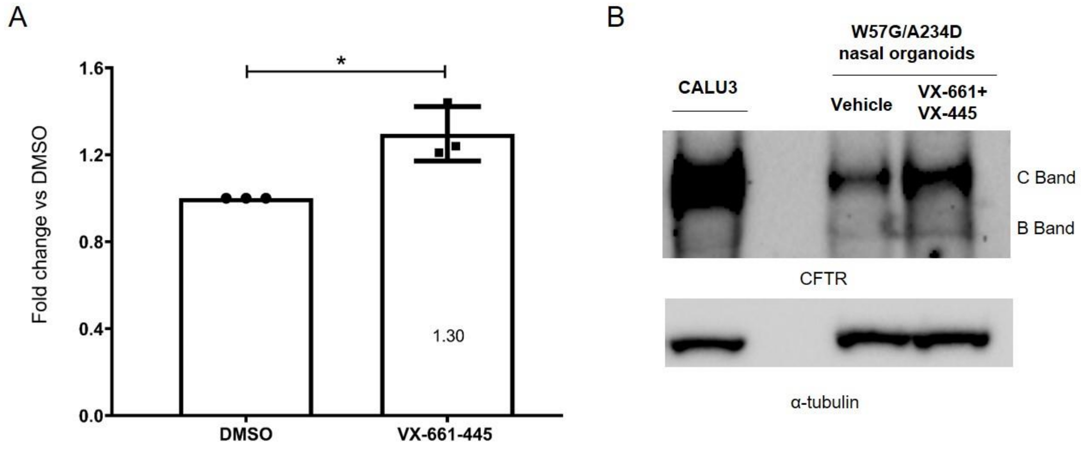

Figure S4. Immunoblot analysis of expression of CFTR protein in W57G/A234D nasal organoid-derived cells, related to Figure 6. A) Densitometric analysis of the results shown as representative western blots in (B). Treatment of nasal organoids with CFTR modulator VX661 in combination with VX445 improved the expression of band C, reaching statistical significance. The results of the densitometric analysis shown in (A) summarize the tubulin-normalized data from three independent experiments. * $p < 0.05$, unpaired t test.

Table S1. Response to CFTR correctors in A234D-CFTR CFBE cells, related to Figure 7. Short circuit current measurement of 4 different clones of CFBE cells expressing CFTR-A234D incubated with DMSO or VX661+VX445 analyzed as described in Han et al. [42].

	DMSO (For/Inh µA)			VX661+VX445 (For/Inh µA)		RNA
**A234D**	**Average**	**Stan Dev**	**WT%**	**Average**	**WT%**	**Average**
**Clone 4 (n≥3)**	9.857	2.534	7.89	69.74	55.83	0.5148
**Clone 6 (n≥3)**	25.82	7.504	29.44	170.02	193.87	0.3614
**Clone 7 (n≥3)**	3.7911	3.3956	4.86	16.53	21.21	0.32115
**Clone 9 (n≥3)**	12.712	5.3418	36.6	26.43	76.07	0.14315
